# Using a Novel Consensus-Based Chemoinformatics Approach to Predict ADMET Properties and Druglikeness of Tyrosine Kinase Inhibitors

**DOI:** 10.3390/ijms262010207

**Published:** 2025-10-20

**Authors:** Evangelos Mavridis, Dimitra Hadjipavlou-Litina

**Affiliations:** Laboratory of Pharmaceutical Chemistry, School of Pharmacy, Faculty of Health Sciences, Aristotle University of Thessaloniki, 54124 Thessaloniki, Greece; eamavridi@pharm.auth.gr

**Keywords:** ADMET properties, tyrosine kinase inhibitors, druglikeness, method validation, molecular docking

## Abstract

The urgent need to reduce the cost of new drug discovery has led us to create a new, more selective screening method using free chemoinformatics tools to restrict the high failure rates of lead compounds (>90%) during the development process because of the lack of clinical efficacy (40–50%), unmanageable toxicity (30%), and poor drug-like properties (10–15%). Our efforts focused on new molecular entities (NMEs) with reported activity as tyrosine kinase inhibitors (small molecules) as a class of great potential. The criteria for the new method are acceptable Druglikeness, desirable ADME (absorption, distribution, metabolism, and excretion), and low toxicity. After a bibliographic review, we first selected the 29 most promising compounds, always according to the literature, then collected the in silico calculated data from different platforms, and finally processed them together to conclude at 14 compounds meeting the aforementioned criteria. The novelty of the present screening method is that for the evaluation of the compounds for Druglikeness, and ADMET properties (absorption, distribution, metabolism, excretion, and toxicity), the data of the different platforms were used as a whole, rather than the results of each platform individually. Additionally, we validated our new consensus-based method by comparing the final in silico results with the experimental values of FDA (Food and Drug Administration)-approved tyrosine kinase drugs. Using inferential statistics of 39 FDA-approved tyrosine kinase drugs obtained after applying our method, we delineated the intervals of the desired values of the physicochemical properties of future active compounds. Finally, molecular docking studies enhance the credibility of the applied method as an identification tool of Druglikeness.

## 1. Introduction

Kinases are a ubiquitous group of enzymes that catalyze the phosphoryl transfer reaction from a phosphate donor to a receptor substrate [1]. There are 518 kinases encoded in the human genome that phosphorylate up to one-third of the proteome. Therefore, kinases have been intensively investigated as potential drug targets over the past 30 years. Virtually every signal transduction process occurs via a phosphor-transfer cascade, indicating that kinases provide multiple nodes for therapeutic intervention in many aberrantly regulated biological processes [2].

A rough classification of major kinases is based on the substrate that they phosphorylate. By adding phosphate groups to substrate proteins, protein kinases are key regulators of cell function, localization, and overall function of many proteins serving to orchestrate the activity of almost all cellular processes.

Protein kinases can be further classified according to their substrate residues as tyrosine kinases, serine/threonine kinases, histidine kinases, and cysteine kinases; more specifically, tyrosine kinases can be classified into receptor and non-receptor protein kinases. Receptor tyrosine kinases (RTKs) are membrane-spanning cell-surface proteins that play critical roles in the transduction of extracellular signals into the cytoplasm. Nonreceptor tyrosine kinases (NRTKs-cytoplasmatic), on the other hand, relay intracellular signals [3]. RTKs and NRTKs transfer a phosphoryl group from a nucleoside triphosphate donor to the hydroxyl group of tyrosine residues on protein substrates, triggering the activation of downstream signaling cascades.

Abnormal activation of tyrosine kinases due to mutations, translocations, or amplifications is implicated in the tumorigenesis, progression, invasion, and metastasis of malignancies. Tyrosine kinase inhibitors (TKIs) are designed to inhibit corresponding kinases [4].

The primary goal of drug discovery and development is to find a molecule with optimal pharmacodynamics, desirable pharmacokinetics, low toxicity, and low synthetic complexity. The pharmaceutical industry faces difficulties achieving this goal, as demonstrated by the high failure rates of lead compounds (>90%) during the development process [5].

Analyses of clinical trial data from 2010 to 2017 show four possible reasons attributed to 90% of clinical failures in drug development: lack of clinical efficacy (40–50%), unmanageable toxicity (30%), poor drug-like properties (10–15%), and lack of commercial needs and poor strategic planning (10%) [6]. Therefore, it is obvious that with the help of silico studies, Druglikeness and ADMET properties are improved to minimize poor pharmacokinetics, adverse toxicity, and, in general, low pharmaco-similarity (overall~45%). Analysis of the observed distribution of some key physicochemical properties of approved drugs, including molecular weight, hydrophobicity, and polarity, reveals that they preferentially occupy a relatively narrow range of possible values. Compounds that fall within this range are described as “druglike.” Note that this definition holds in the absence of any obvious structural similarity to an approved drug [7].

**Figure 1:**
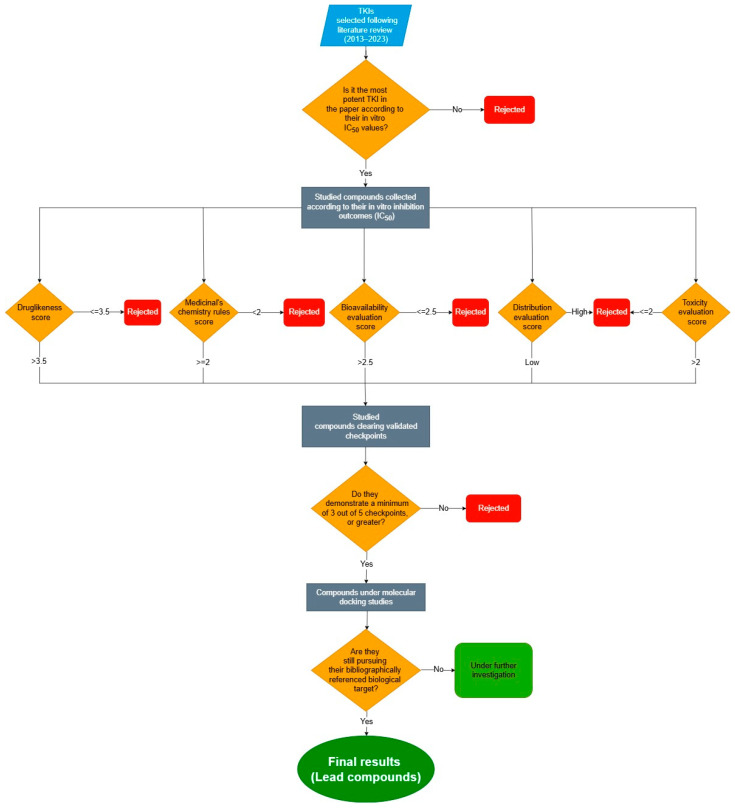
Flowchart describing the procedure of our research.

Following a thorough review of the last decade’s existing literature, we identified newly synthesized small molecules, developed as inhibitors of tyrosine kinases. These compounds either lacked or had minimal in silico studies on their Druglikeness and ADMET properties. In this study, we introduced a new comprehensive approach to assess Druglikeness and ADMET properties, utilizing a combination of data from various computational platforms. We subsequently validated the reliability of this innovative method by comparing its results with experimental data of FDA-approved tyrosine kinase inhibitor drugs, where standalone computational platforms had previously fallen short. Consequently, we evaluated and classified our examined compounds in terms of their acceptable pharmacosimilarity, desirable pharmacokinetics, and low toxicity. The compounds with the highest evaluation scores underwent additional molecular docking analyses using new protocols, to explore the binding patterns to their biological targets as referenced in the literature (Figure 1).

Finally, by leveraging the extensive data gathered through our novel approach for known drug inhibitors, and utilizing suitable statistical methods, we established confidence intervals for essential physicochemical characteristics. This will serve as a crucial resource in future ligand-based virtual screening aiming to discover new potential inhibitors of tyrosine kinases.

## 2. Results and Discussion

Several tools are available and useful to predict in silico Druglikeness and ADMET parameters. In this study, the collected data (last accessed on 24 January 2025) are derived from ten software and web servers (Table 1). The platforms listed are widely recognized and frequently utilized, with many of them cited more than 2000 times on Google Scholar, employing the most recent algorithms. The Toxicity Estimation Software Tool 5.1.2 (T.E.S.T) was created by the United States Environmental Protection Agency (EPA).

Based on the above information, the compounds were identified as follows:

- In terms of complying with known rules of Druglikeness and Medicinal Chemistry such as Lipinski [20], Ghose/CMC-like [21], Veber [22], Egan [5], Muegge [23], MMDR-like [24], Leadlikeness [25], GSK [26], PAINS [27], and Brenk [28];
- In terms of QED parameter (Quantitative Estimate of Druglikeness) [7];
- In terms of pharmacokinetic parameters (Bioavailability, Distribution, and Excretion);
- In terms of toxicity (carcinogenic potential and organ toxicity).

Finally, the above results were quantified, and the compounds were classified to distinguish those that presented the optimal profile (acceptable Druglikeness, desirable pharmacokinetics, low toxicity, and low synthetic complexity).

### 2.1. The Studied Compounds

Through extended bibliographic research conducted from 2013 to 2023, we identified and selected the most active TKI compounds from each study based on their in vitro inhibition results as IC_50_ values ranged from less than 1 nM to 770 nM, with one notable exception at 3200 nM.

As illustrated in Table 2, only 9 out of 29 compounds were subjected to in silico analysis regarding their Druglikeness and ADMET properties, utilizing several platforms including Discovery studio 4.0, QikProp (Schrodinger LLC), SwissADME web tool, and PreADMET 2.0, confirming that our consensus-based method will be beneficial for the initial assessment of the substances.

### 2.2. Calculation/Estimation of Molecular and ADMET Descriptors

For each compound, the average of their molecular descriptors was calculated (Table 3) and tested according to the Druglikeness and Medicinal Chemistry rules (Supplementary Materials, Tables S1 and S2).

The parameters that are directly influenced by the structural and biological variability among the compounds were calculated, since they are essential for evaluating the various Druglikeness rules. We observe that the individual platforms’ data regarding the MW parameter led to comparable results with minor differences, while they produce identical values for the nRbs, nHDr, and MR parameters. A slight yet significant variation is noticed for the nHAc parameter, with the Molsoft platform uniquely calculating TPSA in a different manner. Considerable discrepancies are observed in the estimation of the true value of the Log P_o/w_ parameter, since it is plays a significant role in most Druglikeness models. This variation is correlated to the diverse prediction methods employed across platforms (e.g., ClogP, ALogP, XLogP, MLogP), utilizing different fragment-based or machine learning techniques, leading to varying results. Finally, it is evident that not every platform computes all parameters. Thus, we decided that it would be more suitable to average scores in order to encompass all results.

Accordingly, a qualitative assessment of the ADMET descriptors was performed (Table 4 and Table 5), followed by an overall ADMET evaluation and compounds classification (Supplementary Materials, Tables S3–S5). To enable a comparison among the ADMET descriptors taken from different platforms, we converted all measurements into qualitative estimates according to the explanatory theory underlying each platform. The final assessment is based on the majority principle. In the event of a tie, results from the AdmetLab 3.0, Deep-PK, and admetSAR 3.0 platforms will be considered since they have updated their algorithms more recently.

The classification of P-gp (P-glycoprotein) as a parameter describing Bioavailability or Distribution is still a subject of debate and since, the majority of the platforms listed it under Bioavailability, we followed this convention as well. Furthermore, we did not assess BBB (blood-brain barrier) penetration. Although we gathered the data, we were not focused on the compounds’ biological target specifications at this point.

Considering the toxicological characteristics, we selected the parameters for which it was possible to find a greater amount of experimental data related to approved drugs in order to validate our approach.

### 2.3. Method Validation

To confirm the reliability of the established screening method, we adhered to the protocols used for known tyrosine kinase inhibitor drugs [52], and the compiled findings are presented below (Table 6, Table 7, Table 8, Table 9 and Table 10). Overall, the FDA-approved drugs are assessed, focusing on compounds that attain a score exceeding 50% of the maximum, which seems to be safe.

The last acceptable compound is Mobocertinib, with a score of 3.674. It is evident that some FDA-approved drugs do not meet fundamental Druglikeness criteria, highlighting that establishing our threshold at 50% of the peak value leads us toward more secure outcomes.

In order to meet the criteria for Medicinal Chemistry (the last acceptable drug is Vandetanib), it is essential to exclude undesirable functionalities (such as chemical groups that are recognized as toxic, unstable, or causing false-positive results in biochemical tests), to possess lead-like characteristics, and to maintain a level of simplicity.

For the final determination of Bioavailability, the individual contributions of Caco-2 Permeability, Human Intestinal Absorption, MDCK Permeability, and Pgp-substrate/inhibitor were considered. P-glycoprotein (P-gp), a drug efflux pump, affects the bioavailability of therapeutic drugs and plays a potentially important role in clinical drug–drug interactions. The classification of candidate drugs as substrates or inhibitors of carrier proteins is of crucial importance in drug development. However, regarding the bioavailability of each compound by itself, the best combination is neither an inhibitor nor a substrate, while the worst is a substrate and not an inhibitor. The outcome is under investigation in any intermediate situation. Compounds above Tucatinib were considered to have a high bioavailability.

The low binding of the compound to plasma proteins is an advantageous characteristic allowing quicker results and effectiveness, since only the unbound form of the drug is active pharmacologically. Enhanced tissue penetration is achieved, along with a reduced risk of interactions with other drugs that are highly protein-bound, which may displace it and elevate free drug levels, leading to increased toxicity. However, when clearance is compromised (in cases of kidney or liver disease), the risk of toxicity is enhanced.

For cytotoxic drugs (chemotherapy agents), in terms of the clearance parameter, a high excretion rate is generally preferable to limit toxicity. It should be noted that the ideal balance is affected by various factors, so we cannot determine an optimal value for total clearance.

Upadacitinib is the last compound approved from our method, with a scoring index of 3. Herein, it is important to clarify that our current study does not aim to link scaffolds to score outcomes or suggest structural changes. We focus solely on presenting a new consensus-based screening approach for assessing Druglikeness and ADMET properties, highlighting the most promising compounds.

The in silico derived results of the molecular descriptors were then compared with the experimental data using a simple linear regression model that estimates the relationship between an independent (experimental) and a dependent (predicted) variable using a straight line. Root Mean Squared Error (*RMSE*) and Mean Absolute Error (*MAE*) are metrics used to evaluate a regression model. These metrics tell us how accurate our predictions are and what the amount of deviation from experimental values is. Errors are the differences between the predicted and the experimental values of a variable. There is a third metric—R-Squared score, usually used for regression models. This measures the amount of variation that can be explained by our model, i.e., the percentage of correct predictions returned by our model.

(1) $$RMSE=\sqrt{\frac{1}{N}\sum_{i=1}^{N}{\left(yi-\hat{yi}\right)}^{2}}$$

(2) $$MAE=\frac{\left|yi-\hat{yi}\right|}{N}$$

(3) $$R^{2}=1-\frac{\sum_{i=1}^{N}{(yi-\hat{yi)}}^{2}}{\sum_{i=1}^{N}{\left(yi-\bar{y}\right)}^{2}}$$

The simplest possible “model” is to always predict the mean of *yi* ($\bar{y}$) for all inputs. —The baseline represents the worst acceptable performance. Beating it confirms your model adds value. The *RMSE* of this baseline is:

(4) $${RMSE}_{baseline}=\;\sqrt{\frac{1}{N}\sum_{i=1}^{N}{\left(yi-\bar{y}\right)}^{2}}$$

where *N* is the number of samples, $\hat{yi}$ and *yi* are the predicted and experimental values of the *i*th sample in the dataset; $\bar{y}$ is the mean value of all the experimental values.

Furthermore, the descriptive values were assessed against the experimental findings, leading to models that were evaluated based on their sensitivity (*SE*), specificity (*SP*), and accuracy (*ACC*).

(5) $$SE=\frac{TP}{TP+FN}$$

(6) $$SP=\frac{TN}{TN+FP}$$

(7) $$ACC=\frac{TP+TN}{TP+TN+FP+FN}$$

where *N* is the number of samples, and *TP*, *FP*, *TN*, and *FN* represent true positive, false positive, true negative, and false negative, respectively.

#### 2.3.1. Validation Results of Consensus-Based Model

Initially, experimental data for FDA-approved drugs were collected [53,54,55,56,57] to study the correlation degree with the corresponding in silico data. Data from DrugBank [56] were also utilized (Table 11) in cases where experimental information was lacking. The regression analysis results are presented in Table 12.

In each case (MW, TPSA, and MR), Pearson correlation coefficient (r) values of 1.000, 0.995, and 0.950, were determined, respectively. These figures indicate a robust correlation with the values calculated from DrugBank. Additionally, the error variation (mean values derived from *RMSE* and *MAE*) is approximately 0.2 units for MW, 2.9 units for TPSA, and 6.4 units for MR, which are relatively small when compared to the means of the samples, also highlighting a strong relationship between the calculated values from DrugBank and the consensus-predicted figures. Comparing *RMSE* to *RMSEbaseline*, we found that the performance of our models shows improvements of 99.68%, 85.80%, and 66.93%, respectively, over the baseline, indicating a reduction in errors.

In the case of the LogP_o/w_ parameter (Table 13), however, we observed that r < 0.700 (R = 0.645); thus, we decided to use an alternative approach. The measurement averaging from Molinspiration–Molsoft platforms showed the best coefficient, r = 0.750, compared to any other case.

The error variation is about 1.00 units for the initial case and 0.89 units for the second, indicating a slight enhancement, though it remains relatively high when compared to the sample means. Nevertheless, our final model offers a statistically significant improvement over the baseline, achieving a 32.6% reduction in errors, unlike the 20.2% reduction given in the first model. As stated previously, estimating LogP_o/w_ is a challenging task for various reasons; primarily, LogP_o/w_ is influenced by pH (particularly for ionizable compounds), and most predictive models still assume that the compounds are neutral. In addition, conformational flexibility and other factors contribute to increased variability in experimental LogP_o/w_ measurements. Considering all these factors, our consensus model is currently accepted until a more effective solution is developed.

We did not consider the R^2^ parameter at all, because it indicates the percentage of correct predictions returned by the regression equation; however, this is not relevant to us since our primary interest is related to measuring the evaluation parameters between the predicted values ($\hat{yi}$) and the “real” values (*yi*).

We confirmed the validity of MW, LogP, TPSA, and MR, which are affected by biological variability. Furthermore, we gathered information on nHA, nHD, nRing, and nRigidB, which are 2D descriptors; however, these parameters did not show any considerable variation across platforms. Thus, it seemed less important to be compared to “real” data.

**Figure 2:**
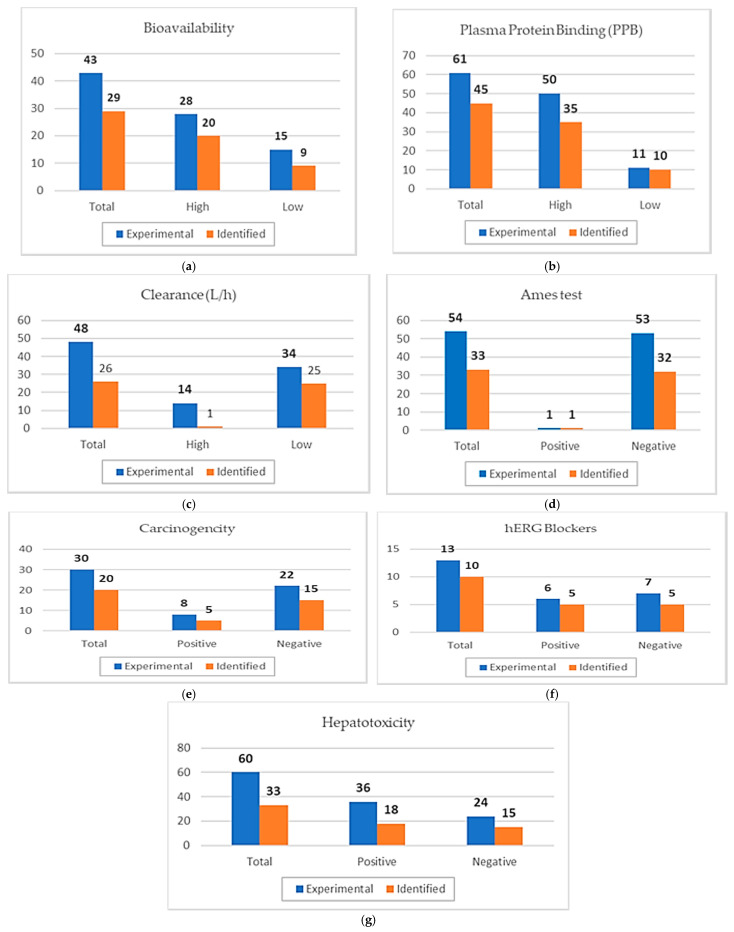
Bar graphical representation of: (**a**) Bioavailability; (**b**) Plasma Protein Binding; (**c**) Clearance; (**d**) Ames test; (**e**) Carcinogenicity; (**f**) hERG; (**g**) Hepatotxicity assessments, showing Experimental Total = *N*, Identified high/positive = *TP*, Identified low/negative = *TN*, experimental low/negative − identified low/negative = *FP*, experimental high/positive − identified high/positive = *FN*.

Moving to ADMET descriptors’ validation of reliability (Table 14), we have drawn the following graphs to test the ability of our method in order to detect experimental values (Figure 2).

Applying Equations (5)–(7) to each parameter, we found that the bioavailability model presents sensitivity (*SE*) = 0.714, specificity (*SP*) = 0.600, and accuracy (*ACC*) = 0.674, and the PPB model exhibits *SE* = 0.700, *SP* = 0.909, and *ACC* = 0.738, confirming both as suitable predictive models. However, the Clearance parameter is significantly underestimating true positive results with a *SE* = 0.0714, although it presents acceptable *SP* = 0.600 and *ACC* = 0.674. Regarding Toxicity, all individual parameters qualify as reliable the models, with the Ames test showing *SE* = 1.00, *SP* = 0.604, = *ACC* of 0.611; Carcinogenicity displaying *SE* = 0.625, *SP* = 0.682, and *ACC* = 0.667; hERG Blockers presenting *SE* = 0.833, *SP* = 0.714, and *ACC* = 0.769; and Hepatotoxicity yielding *SE* = 0.500, *SP* = 0.625, and *ACC* = 0.550. Considering that any value above 0.500 is deemed satisfactory for the reliability of our consensus model, only the Clearance parameter is excluded and thus cannot be incorporated into our screening method. Clearance, which pertains to excretion, is a vital pharmacokinetic parameter for assessing the behavior of drugs within the body; however, it is not essential for defining the pharmacosimilarity of a promising compound. Consequently, the success of the Bioavailability, Distribution, and Toxicity models mitigates the setback of the Excretion deficiency.

#### 2.3.2. Validation Results of Individual Platforms

The same approach was primarily utilized for four distinct platforms, namely AdmetLab 3.0, pkSCM, Deep-PK, and admetSAR 3.0, as they compute most of the requested parameters (Table 1). In the physicochemical predictions, all platforms performed well with the exception of Log P_o/w_, which was evaluated through all platforms; each one failed to be compared to our consensus-based model, with Pearson correlation coefficients ranging from 0.314 (AdmetLab) to 0.734 (Molsoft) (Supplementary Materials, Figure S1). Regarding ADMET descriptors, our model outperformed each individual platform in terms of Bioavailability, Ames test, and Carcinogenicity, achieving similar results to Deep-PK in Hepatotoxicity. AdmetLab demonstrated marginally better performance in Distribution (*SE* = 0.880, *SP* = 0.818, *ACC* = 0.869) and hERG Blockers (*SE* = 1.000, *SP* = 0.857, *ACC* = 0.923) but did not exceed the threshold of 0.500 for any other parameter. No individual platform or consensus estimation produced satisfactory results in plasma clearance. This could be related to inadequate training of the algorithms used in the platforms. Thus, we decided to not consider our screening method on excretion parameters (Supplementary Materials, Figure S2).

### 2.4. Evaluation of Druglikeness, Medicinal Chemistry, and ADMET Properties

In Table 15 are the compounds that were excluded from the validated screening method following the defined criteria: Druglikeness, Medicinal Chemistry, Bioavailability (ADME), Distribution (ADME), and Overall Toxicity (Toxicity). As mentioned before, for each individual parameter, the acceptance threshold was set at >50% of the maximum score, whereas for the Distribution, only compounds with low plasma protein binding were selected.

A total of 24 compounds exceeded the Druglikeness threshold of 3.501, while 16 were rated above the acceptable medicinal chemistry score of 2. Regarding Bioavailability, 8 out of 29 examined compounds did not achieve a value greater than 2.51. In terms of Distribution, only compounds with low PPB were evaluated, and when it comes to Overall Toxicity, nine compounds achieved the minimum acceptable score of 2.01 (Supplementary Materials, Tables S1–S5).

After the excluded compounds were presented, they were arranged based on the number of hits in the table. Dark green was assigned for 5/5 hits, light green for 4/5, yellow for 3/5, red for 2/5, and white for 1/5.

Compounds that demonstrate greater than three out of five hits (≥60%) were considered appropriate (Table 16).

It is evident that Distribution is the most frequently violated property. Only two compounds among our compounds’ pool were identified to have low PPB. However, as illustrated in Table 9, a significant portion of FDA-approved drugs are actually found experimentally to exhibit high PPB; therefore, low PPB does not exclude a compound from being classified as Druglike. Additionally, Overall Toxicity is listed as the second most frequently violated property, attributed to the mechanism of action of TKIs, since they inhibit cell growth and division.

Following the application of our approach to FDA-approved drugs, 39 of the 63 compounds met the necessary criteria and were subsequently utilized in the ensuing statistical analyses.

### 2.5. Molecular Docking Studies

The compounds that were highlighted during the screening phase proceeded to molecular docking studies, initially focusing solely on their primary biological targets, already reported in the literature [29,30,31,32,33,34,35,36,37,38,39,40,41,42,43,44,45,46,47,48,49,50,51]. Consequently, the studied biological targets include **VEGFR-2**, **RET**, **MET**, **EGFR-HER-1**, **HER-2**, and **BTK**. It is important to mention that TKI.4 was identified as a strong and highly selective small molecule inhibitor of c-MET, known as Tepotinib, which has been authorized for the treatment of advanced lung cancer in patients with specific genetic mutations. This also indicates the reliability of the computational methods and techniques used in the study. The remaining compounds that retained their initial biological focus consist of **TKI.2a**, **TKI.2b**, **TKI.19** (biological target identified: VEGFR-2), **TKI.6** (biological target identified: HER-2), and **TKI.21b** (biological target identified: EGFR). Molecular docking studies were performed and the binding efficiency of our compounds was evaluated by (i) measuring their binding affinity (kcal/mol), (ii) calculating the possibility that the pose displays a minimal Root Mean Square Deviation (RMSD) to the binding pose (CNN pose score), (iii) assessing their affinity to the biological target as determined by the CNN (CNN affinity), and, naturally, (iv) examining the interactions present in docking complexes.

**Figure 3:**
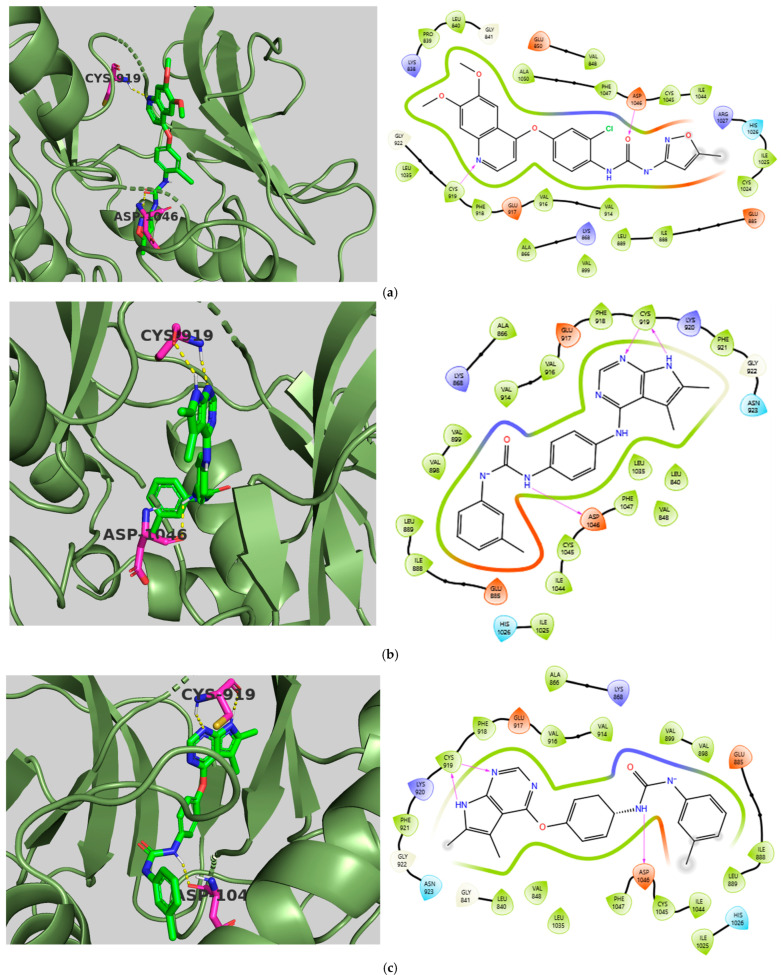

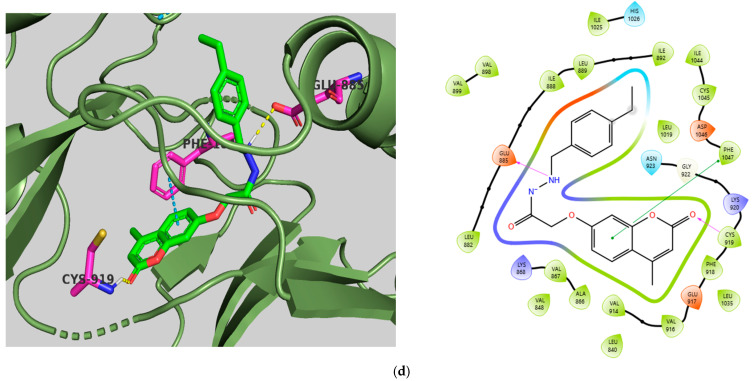
Preferred docking pose (3D) and ligand interaction diagram (2D) of (**a**) Tivozanib; (**b**) TKI.2a; (**c**) TKI.2b; (**d**) TKI.19, with VEGFR-2 (PDB ID: 4ASE). Green core: Ligands; Magenta core: Key amino acids; Yellow dashes/Magenta arrows: Hydrogen bond interactions; Cyan dashes/Green line: π-π interactions.

The chosen X-ray crystal structure of VEGFR-2 (PDB ID: 4ASE—Tivozanib) [58] displays a ‘DFG-out’ (inactive) conformation, indicating that the kinase is primarily inactive, as the DFG (Asp-Phe-Gly) residues are oriented in a manner preventing ATP binding and obstructing the substrate binding site. The active site of VEGFR-2 is divided into four key regions: the hydrophobic regions (HYD-I and HYD-II), a hinge region, and the DFG motif region. The HYD-I region serves as the active site where ATP and type I inhibitors bind selectively, while the HYD-II region, known as the ‘Phe pocket’ or ‘allosteric site,’ is where most type II inhibitors bind specifically. Consequently, Tivozanib, as a type II inhibitor, interacts specifically with the ‘DFG-out’ conformation, thus cannot bind to the ATP binding pocket and instead binds to the receptor’s adjacent hydrophobic site. Among type I (first-generation) and type II (second-generation) inhibitors, type II inhibitors associated with the ‘DFG-out’ conformation have shown advantages regarding selectivity and off-target activity (side effects) [59]. Regarding the docking results, all three compounds displayed binding affinities and CNN pose scores ranging from −10.33 to −11.94 kcal/mol and 0.855 to 0.869, respectively, which are comparable to the co-crystallized ligand, Tivozanib, with a binding affinity of −10.87 kcal/mol and a CNN pose score of 0.925. For CNN affinity, TKI.2a (8.068) and TKI.2b (8.030) reported values that are similar to Tivozanib (8.124), except for TKI.19 (7.055). The ligand Tivozanib, when redocked in its co-crystal form, established two hydrogen bonds: one with the hinge region at Cys919 and the other with Asp1046 in the DFG domain. Hydrophobic interactions were noted in the HYD-I region with Leu840 and Phe918, as well as in the more selective HYD-II region involving Ile888, Leu889, Val899, and the gatekeeper residue Val916. Additionally, all three studied compounds exhibited H-bond interactions with Cys919; particularly, TKI.2a and TKI.2b formed two hydrogen bonds, critical for the molecule’s inhibitory activity. Regarding the DFG motif region, TKI.2a and TKI.2b showed hydrogen bonding with the amino acid Asp1046 through the urea moiety, similar to Tivozanib, while TKI.19 interacted with Glu885, another constituent of the DFG domain, through its carboxamide group. This variation may be a reason for TKI.19’s poorer performance in the CNN affinity score. Common hydrophobic interactions were observed in the HYD-I region among all compounds (Leu840, Val848), while slight variations were present in the HYD-II region between TKI.2a, TKI.2b (Leu889, Val899, Phe1047), and TKI.19 (Leu889, Val898, Ile1044). Furthermore, TKI.19 exhibited a π-π interaction with Phe1047 (Figure 3).

The HER kinase family, also known as the human epidermal growth factor receptor (HER) or epidermal growth factor receptor (EGFR), comprises four members: EGFR (HER1 or ErbB-1), HER2 (ErbB-2 or neu), HER3 (ErbB-3), and HER4 (ErbB-4). These are multidomain proteins that include an extracellular domain for ligand binding, a single transmembrane domain, and an intracellular domain with tyrosine kinase activity. In normal tissues, ERBB signaling begins when ligands bind to the extracellular domains of EGFR, HER3, or HER4, leading to either homo- or heterodimerization [60].

**Figure 4:**
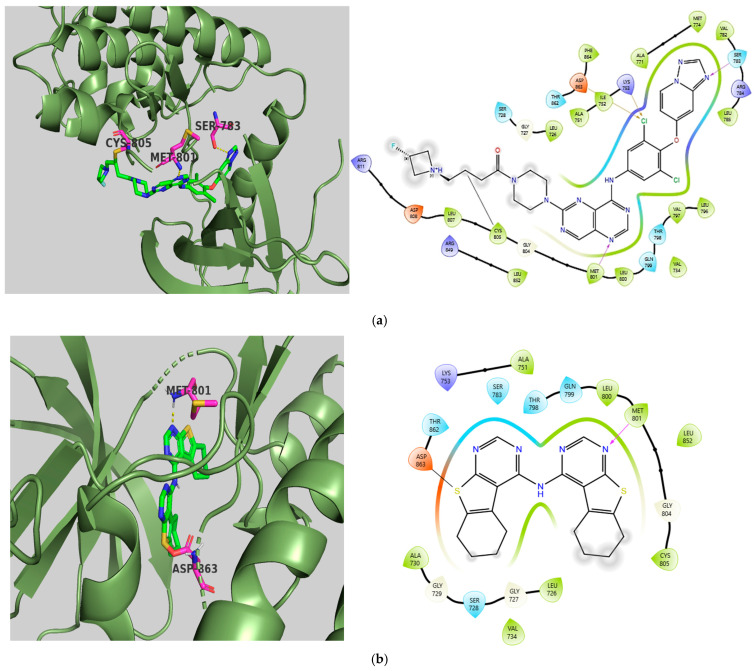
Preferred docking pose (3D) and ligand interaction diagram (2D) of (**a**) covalent inhibitor; (**b**) TKI.6, showing key interactions at the active site of 7PCD. Green core: Ligands; Magenta core: Key amino acids; Yellow dashes/Magenta arrows: Hydrogen bond interactions; Orange arrows: Halogen bonds; Black lines: Covalent bonds.

Unlike the other members, HER2 is not activated by ligands; instead, it serves as the preferred dimerization partner for the other ERBB family members. The selected X-ray crystal structure of HER2 (PDB ID: 7PCD—covalent inhibitor) [60] exhibits the characteristic bilobed folding of kinases. The two lobes are linked by a flexible hinge region and divided by a deep cleft that contains the ATP binding site [61]. Docking studies of TKI.6 revealed that a significant hydrogen bond formed at the hinge region with Met801, similarly to the co-crystalized inhibitor. Furthermore, while the integrated ligand showed a covalent bond with cysteine at position 805, our compound was permanently linked to Asp863 in the DFG motif domain. Comparable binding affinities were observed between the covalent inhibitor (binding affinity: −9.66 kcal/mol, CNN affinity: 7.734) and TKI.6 (binding affinity: −8.96 kcal/mol, CNN affinity: 7.684), emphasizing that the formation of a covalent bond between the inhibitor and the protein enhances binding affinity and potency. The variance in CNN pose scores between the co-crystallized inhibitor (0.814) and TKI.6 (0.920) could be attributed to the differing binding residues in the ATP binding site, indicating that the compound we studied may have a higher likelihood of adopting a favorable pose. Additionally, hydrophobic interactions with the glycine-rich nucleotide phosphate-binding loop (Leu726, Val734) contribute to the stability of the complex. Finally, although the serine located at position 783 is considered an important selectivity-determining amino acid between HER2 and EGFR activity, no interactions were observed (Figure 4).

**Figure 5:**
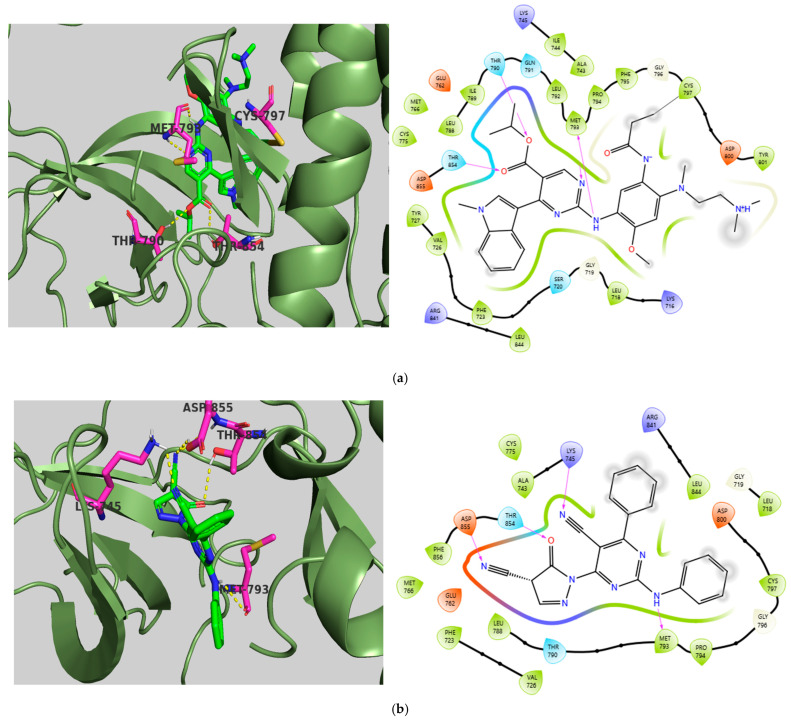
Preferred docking pose (3D) and ligand interaction diagram (2D) of EGRF with (**a**) Mobocertinib; (**b**) TKI.21b, showing key interactions at the active site of 7T4I. Green core: Ligands; Magenta core: Key amino acids; Yellow dashes/Magenta arrows: Hydrogen bond interactions; Black lines: Covalent bonds.

As our molecular docking research focused on another member of the HER kinase family, namely EGFR, we utilized the surrogate crystal structure of the wild-type EGFR complexed with Mobocertinib (PDB ID: 7T4I) [62]. This research highlights important residues, including Met793 found in the hinge area, as well as specific regions like the selectivity pocket where Thr790 functions as the “gatekeeper” for ATP binding, Lys745 serves as the catalytic lysine, and Thr854 is situated in the DFG triad, along with two separate hydrophobic regions. The hydrophobic region I consists of amino acids such as Phe723, Leu747, Ile759, Met766, Leu777, and Leu788, while hydrophobic region II, located near Thr790 and comprising Leu718, Gly719, Val726, and Leu844, plays a crucial role in the binding of compounds to EGFR. Lastly, Cys797, positioned at the edge of the active site cleft and being the most solvent-exposed cysteine in the EGFR kinase domain, is responsible for forming covalent bonds with irreversible TKIs [36,63]. Both the co-crystalized ligand and TKI.21b formed hydrogen bonds with the hinge region (Met793) and the DFG motif (Thr854). However, Mobocertinib demonstrated better positioning in the active site compared to TKI.21b due to an additional hydrogen bond with Met793, another hydrogen bond with the “gatekeeper” Thr790, and a covalent bond with Cys797, which accounts for the disparity in their CNN pose scores of 0.970 and 0.860, respectively. Conversely, TKI.21b established hydrogen bonds with catalytic residue Lys745 and Asp855, along with significant hydrophobic interactions involving Leu718, Phe723, Val726, and Leu844, leading to comparable binding affinities with Mobocertinib (TKI.21b binding affinity: −8.25 kcal/mol, CNN affinity: 7.493; Mobocertinib binding affinity: −7.66 kcal/mol, CNN affinity: 8.106) (Figure 5).

#### Molecular Docking Validation Results

Common methods for assessing the accuracy of docking protocols include self-docking, cross-docking, and ligand enrichment. Self-docking is a highly employed technique for the preliminary evaluation of a docking program’s accuracy. As a validation technique, it aims to reproduce the original orientation of the co-crystallized ligand, whereas cross-docking assesses how well a particular receptor positions chemically diverse groups of ligands while maintaining acceptable RMSD values [64]. In the process of enriching the database, decoys are introduced among a group of active inhibitors, and the docking software is evaluated for its effectiveness in ranking the active substances. The decoy molecules mimic the active compounds by sharing similar physical characteristics; however, they must have no binding affinity for the receptor.

Docking setup was first validated by re-docking of the co-crystallized ligand in the vicinity of the binding site of the enzyme, followed by calculating the Root Mean Square Deviation (RMSD) between the final configuration and the initial coordinates. RMSD values below 2.0 Å signify consistent results, values between 2.0 Å and 3.0 Å indicate a shift from the reference position while keeping the desired orientation, and RMSD values exceeding 3.0 Å are entirely inaccurate [65]. Regarding our molecular docking investigations, we achieved reliable RMSD values for VEGFR-2 of 1.144 Å, for HER2 of 1.121 Å, and for EGFR of 1.430 Å, all remaining below 1.5 Å.

To further assess the docking protocols, cross-docking procedures were implemented. In the cross-docking analysis, each known FDA-approved ligand of the specified biological targets was docked into the receptors mentioned (PDB IDs: 4ASE, 7PCD, and 7T4I). According to Table 17, a significant majority (11 out of 18) of the known drugs achieved measurements below 2.5 Å, with 4 of them falling between 2.5 and 3 Å, confirming the reliability of our docking protocols. The only exceptions were Axitinib, Pazopanib, and Sunitinib in their molecular studies on VEGFR-2, which exhibited measurements greater than 3 Å. Notably, these three compounds share a common characteristic of demonstrating low affinity, CNN pose score, and CNN affinity values collectively.

**Figure 6:**
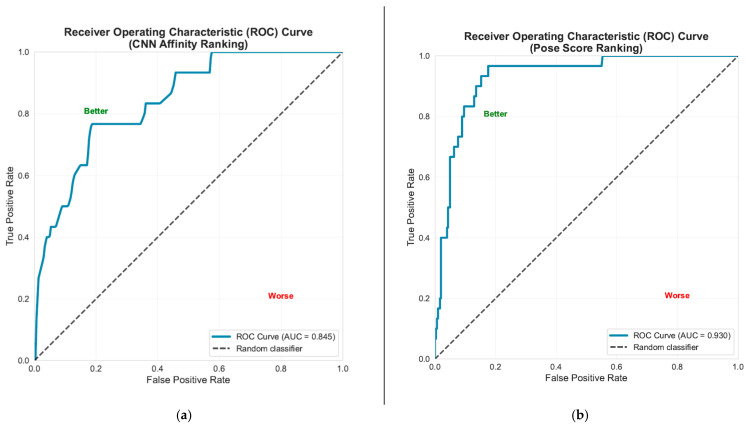
Receiver operating characteristic (ROC) curve and area under the ROC curve (AUC-ROC) ranked by (**a**) CNN affinity; (**b**) CNN pose score.

The enrichment factor (*EF*) serves as a measure of the docking program’s reliability. The objective was to evaluate the ability of the receptor to differentiate between inactive substances and known active compounds by determining enrichment values. The enrichment factor was computed using the formula below [66]:

(8) $${EF}_{X\%}=\frac{\frac{{actives}_{x\%}}{{dataset}_{x\%}}}{\frac{{actives}_{total}}{{dataset}_{total}}}$$

where *actives_x_*_%_ refers to the active compounds present in the selected dataset (*dataset_x_*_%_), while *dataset_total_* encompasses all compounds within that dataset, and *actives_total_* indicates the number of active molecules included among the decoys. We defined *x*% as 10%, which means we aimed to determine how many active compounds exist within the top 10% of our ranked dataset. An enrichment factor (*EF*) exceeding 1 demonstrates that the approach is more efficient than random selection, with higher values indicating improved performance. For instance, an *EF* of 5 in the top 10% of the dataset implies that there are five times more active compounds present in that top 10% of the evaluated set than one would anticipate by random chance. Herein, we employed a HER2 protein (PDB ID: 3PP0) as the receptor, utilizing a dataset of 332 compounds that included 30 active compounds among 302 inactive ones. As a result, when ranking compounds based on their CNN affinity values, the enrichment factor at 10% (*EF*(10%)) was found to be 4.360, identifying 13 active compounds in the top 33 structures (which represents 10%) (Supplementary Materials, Table S6), while ranking based on the CNN pose score yielded an *EF*(10%) of 6.036, highlighting 18 actives within the top 33 structures (Supplementary Materials, Table S7). Moreover, the Receiver Operating Characteristic (ROC) Curve and the Area Under the Curve (AUC) provide valuable insights into the model’s capacity to differentiate between active and inactive compounds across various threshold settings, with the AUC serving as a summary of the model’s overall performance, as illustrated in Figure 6. It is evident that the ranking based on the CNN pose score exhibited a higher ROC-AUC compared to the CNN affinity ranking, with values of 0.930 and 0.845, respectively, indicating that utilizing the CNN pose score for ranking is a more dependable approach.

Reviewing all the docking validation parameters presented, it is confirmed that the used docking software and protocols reliably produce consistent and trustworthy results.

### 2.6. Statistical Results

A confidence interval refers to the probability that a population parameter will be found between a set of values for a certain proportion of times. Statisticians use confidence intervals to measure the uncertainty in an estimate of a population parameter based on a sample. Therefore, by calculating the confidence limits (a = 0.05) of the molecular descriptors for the distinguished FDA-approved drugs, we estimated the range within the true population mean (the true mean of all active tyrosine kinase inhibitors), is likely to lie.

Data obtained from the independent T-tests that we performed for each molecular descriptor of the two samples (studied compounds and FDA-approved drugs) (Table 18) (Supplementary Materials, Table S8) confirmed that mean values of the molecular descriptors of the studied compounds fall within the confidence limits of the corresponding molecular descriptors of FDA-approved drugs, with the exception of the mean value of the LogP_o/w_ parameter.

Alongside analyzing the average values of the two datasets, we conducted a Kolmogorov–Smirnov (KS) test to evaluate the distributions of the datasets (Table 19) (Supplementary Materials, Figure S3).

Therefore, the above data suggests that we cannot reject the null hypothesis, e.g., that the molecular descriptors of the two different samples come from the same population (*p* > 0.05), except for the LogP_o/w_ parameter (*p* < 0.05). One possible reason for the deviation in the LogP_o/w_ value could be that FDA-approved drugs have undergone lead optimization to achieve a balanced LogP_o/w_ for ADME, while our compounds may still be in an earlier development stage where LogP_o/w_ has not been refined.

These findings are important because, from different samples, we can use inferential statistics to draw some conclusions about the molecular descriptors of the general population, and we will be able to more accurately approximate the molecules that are worth investigating as tyrosine kinase inhibitors.

## 3. Materials and Methods

In this study, we evaluated the Druglikeness and ADMET characteristics of various TKIs sourced from the literature [29,30,31,32,33,34,35,36,37,38,39,40,41,42,43,44,45,46,47,48,49,50,51], aiming to identify the most promising and effective tyrosine kinase inhibitors with acceptable pharmacosimilarity, favorable pharmacokinetics, and minimal toxicity. To address this, we created a new consensus-based screening approach that leverages 10 well-regarded free chemoinformatics platforms, specifically Molinspiration [8], Molsoft [9], SwissADME web tool [10], Mcule [11], AdmetLab 3.0 [12,13], pkSCM [14], Deep-PK [15], admetSAR 3.0 [16,17], PreADMET 2.0 [18], and T.E.S.T 5.1.2 [19]. The forecasted data was compiled in Tables S1–S5 (Supplementary Materials).

Additionally, we conducted a comparative analysis using data from 63 FDA-approved TKIs. Bibliographic references were gathered from R. R. Shah et al., 2013 [53], J. Dulsat et al., 2023 [54], M. Viganò et al., 2023 [55], C. Knox et al., 2024 [56], and the U.S. Department of Health and Human Services Food and Drug Administration [57], and are displayed in Table 11 and Table 14. Nevertheless, for numerous compounds, we could not locate experimental data for the in silico predicted ADMET characteristics, such as skin permeability, skin sensitization, and ecological toxicity, leading us to exclude these properties from our screening method.

In molecular docking studies, the X-ray crystal structures were retrieved from the Protein Data Bank on the Research Collaboration for Structural Bioinformatics (RCSB) website www.rcsb.org (last accessed on 2 July 2025). The preparation of proteins was performed using OpenMM 8 [67]—energy minimizations were executed with the AMBER 14 or Charm36 force fields—while GypSUm-DL [68] was used to generate and minimize ligand 3D coordinates. Docking was conducted using GNINA 1.0 [69], a molecular docking tool that incorporates convolutional neural networks (CNNs) for scoring and optimizing ligands. The input files for docking were visualized using PyMOL 3.0.4 [70] and Schrödinger Maestro 14.5.131 [71]. For the validation of protocols related to molecular docking studies (including re-docking and cross-docking), we utilized suitable Python 3.2.2 scripts to identify matching atoms between the docked ligands and the co-crystallized ligands and finally compute the RMSD values. In terms of verifying the docking software, the ultimate goal involved using active structures and decoys sourced from a publicly accessible repository (InformaticsMatters, 2021) [72]. Additionally, ROC-AUC curves were created using appropriate Python scripts.

The in silico data of the FDA-approved drugs obtained from the studies were statistically analyzed using IBM SPSS Statistics (Version 29) [73]. Thus, for each physicochemical property, the confidence intervals for a 95% confidence level (i.e., corresponding significance level of 0.05 or 5%) were calculated. Finally, the two samples (39 FDA-approved drugs and 29 studied compounds) were subjected to a T-test. This analysis compares the average values of two datasets, in relation to a non-parametric Kolmogorov–Smirnov (KS) Test, which investigates if two datasets are taken from the same distribution, in order to determine if they came from the same population. The comparative statistical data along with the data tables can be found in Table 18, Table 19 and Table S6.

## 4. Conclusions

The novelty of the present screening method is that for the evaluation of the compounds for Druglikeness and ADMET properties, the data from the different platforms were used as a whole, rather than the results of each platform individually. After all, according to Stephen Hawking, ‘Science is beautiful when it makes simple explanations of phenomena or connections between different observations. Examples include the double helix in biology and the fundamental equations of physics.’ The reliability validation of the new method showed that it could approximate experimental values, in contrast to individual platforms validation results, unlike the validation results derived from the individual platforms.

Out of the 29 compounds listed in Table 2 from the literature, 14 compounds shown in Table 16 satisfied more than 60% of the criteria we established: Druglikeness, Medicinal Chemistry rules, Bioavailability, Distribution, and Overall Toxicity. Notably, **TKI.4, TKI.16, TKI.19, TKI.21b, AIK.1, and DDK.8** exhibited the most favorable profiles. In our initial molecular docking studies, we discovered that TKI.4 is the approved drug Tepotinib, which enhances the credibility of our consensus-based method as a tool for identifying drug-like substances. Among all the other compounds, only TKI.2a, TKI.2b, TKI.19 (VEGFR-2), TKI.6 (HER2), and TKI.21b (EGFR) successfully confirmed their original biological target designation, as indicated by our newly validated molecular docking protocols that employ deep learning models for evaluation and ranking.

In addition, using inferential statistics, we demonstrated that we are 95% confident that the mean values of the molecular descriptors, for the set of active small molecules potentially acting as tyrosine kinase inhibitors, are within the confidence limits calculated from the FDA-approved drugs that picked/selected out from our screening method. These limits were defined as follows: **MW [416.81, 461.47], TPSA [83.48, 95.83], MR [115.14, 128.65], LogPo/w [2.62, 3.49], nRB [5, 7], nHA [7, 8], nHD [2, 2], nRings [4, 5], nRigidB [23, 26], and nAtoms [52, 58]**.

At this point in our investigation, our main objective was to present a new screening method rather than to analyze the results for their possible biological significance. This will be addressed in our future work, utilizing suitable chemoinformatics tools.

Noting that the Druglikeness definition holds in the absence of any obvious structural similarity to an approved drug [7], we will aim in the future to uncover structural similarities among the selected compounds we studied and FDA-approved tyrosine kinase inhibitors by conducting molecular similarity studies. This will serve both as a manifestation of Druglikeness and as a tool to discover additional biological targets [74]. Furthermore, to assess the biological significance of proposed or newly identified targets, we will perform molecular docking studies and pharmacophore modeling, with the ultimate objective of suggesting structural modifications.

## Figures and Tables

**Table 1:** Free web servers (Molinspiration, Molsoft, SwissADME, Mcule, AdmetLab, pkSCM, Deep-PK, admetSAR, PreADMET) and software (T.E.S.T) used to predict physicochemical properties, Bioavailability, Distribution, Excretion, and Toxicity.

**Descriptors**		**Software/Webservers**									
		Molinspiration [8]	Molsoft [9]	SwissADME [10]	Mcule [11]	AdmetLab 3.0 [12,13]	pkSCM [14]	Deep-PK [15]	admetSAR 3.0 [16,17]	PreADMET [18]	T.E.S.T [19]
**Physicochemical Properties**	Molecular weight	✓	✓	✓	✓	✓	✓	✓	✓	✕	✕
	TPSA	✓	✓	✓	✓	✓	✕	✓	✓	✕	✕
	Molar Refractivity	✕	✕	✓	✓	✕	✕	✕	✕	✕	✕
	Log P_o/w_	✓	✓	✓	✓	✓	✓	✓	✓	✕	✕
	Num. rotatable bonds	✓	✕	✓	✓	✓	✓	✓	✓	✕	✕
	Num. H-bond acceptors	✓	✓	✓	✓	✓	✓	✓	✓	✕	✕
	Num. H-bond donors	✓	✓	✓	✓	✓	✓	✓	✓	✕	✕
	Num. Rings	✕	✕	✕	✓	✓	✕	✕	✕	✕	✕
	Num. Rigid bonds	✕	✕	✕	✕	✓	✕	✕	✕	✕	✕
	Num. atoms	✕	✕	✕	✓	✕	✕	✕	✕	✕	✕
**Bioavailability**	Caco-2 Permeability	✕	✕	✕	✕	✓	✓	✓	✓	✓	✕
	Human Intestinal Absorption	✕	✕	✓	✕	✓	✓	✓	✓	✓	✕
	MDCK Permeability	✕	✕	✕	✕	✓	✕	✓	✓	✓	✕
	Pgp-substrate	✕	✕	✓	✕	✕	✓	✓	✓	✕	✕
	Pgp-inhibitor	✕	✕	✕	✕	✕	✓	✓	✓	✓	✕
**Distribution**	Plasma Protein Binding (PPB)	✕	✕	✕	✕	✓	✕	✓	✓	✓	✕
**Excretion**	Total Clearance	✕	✕	✕	✕	✓	✓	✓	✓	✕	✕
**Toxicity**	Mutagenicity (Ames test)	✕	✕	✕	✕	✓	✓	✓	✓	✓	✓
	Carcinogencity (rat)	✕	✕	✕	✕	✓	✕	✓	✓	✓	✕
	hERG Blockers	✕	✕	✕	✕	✓	✓	✓	✓	✓	✕
	Hepatotoxicity	✕	✕	✕	✕	✓	✓	✓	✓	✕	✕

✓ Prediction data were provided; ✕ prediction data were not provided.

**Table 2:** Studied compounds gathered according to their in vitro inhibition outcomes (IC50).

A/A	Compound	Structure	Reported Biological Target	In Vitro Enzyme Inhibition Assay IC50 (nM)	In Silico Studies	Year of Publication	Reference
1	TKI.1	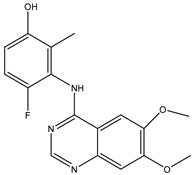	Ret	44	-	2016	[29]
2	TKI.2a	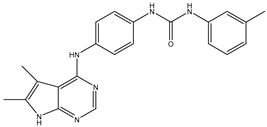	VEGFR-2	11.9	Docking	2018	[30]
3	TKI.2b	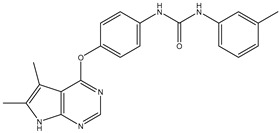	VEGFR-2	13.6	Docking	2018	
4	TKI.3	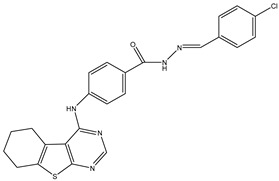	VEGFR-2	230	ADMET /Docking	2021	[31]
5	TKI.4	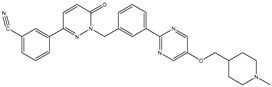	c-Met	<1	Docking	2015	[32]
6	TKI.5	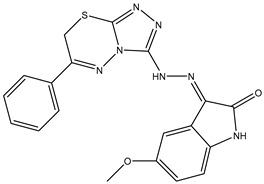	Dual VEGFR-2/C-Met	435/ 654	Docking	2020	[33]
7	TKI.6	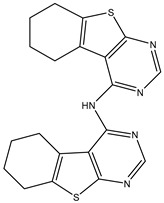	dual EGFR/HER-2	278/ 415	Docking	2019	[34]
8	TKI.7a	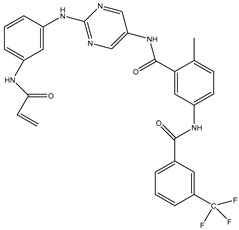	BTK	5	Docking	2013	[35]
9	TKI.7b	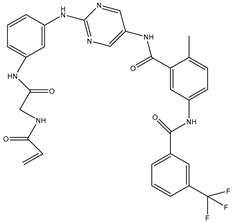	BTK	4.4	Docking	2013	
10	TKI.8	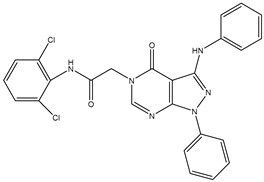	EGFR	97	ADMET /Docking	2021	[36]
11	TKI.9	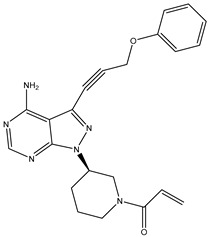	BTK	7.95	Docking	2018	[37]
12	TKI.10	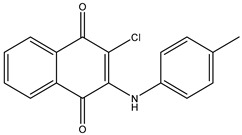	EGFR	3.96	ADMET /Docking	2022	[38]
13	TKI.11	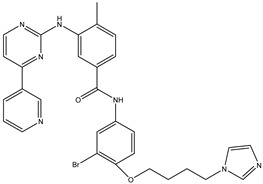	BCR-ABL	37	Docking	2022	[39]
14	TKI.13a	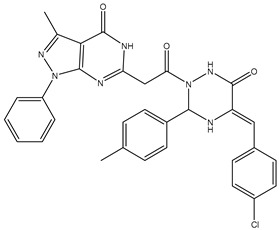	dual EGFR/HER-2	420	ADMET	2014	[40]
15	TKI.13b	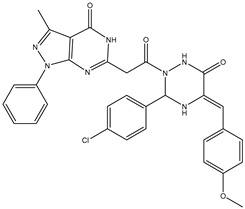	dual EGFR/HER-2	220	ADMET	2014	
16	TKI.14a	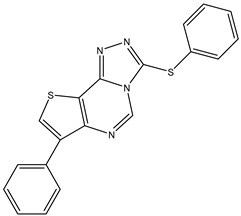	EGFR	147	Docking	2021	[41]
17	TKI.14b	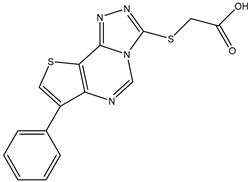	EGFR	185	Docking	2021	
18	TKI.15	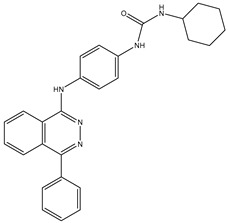	VEGFR-2	140	Docking	2020	[42]
19	TKI.16	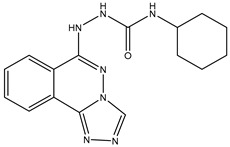	VEGFR-2	110	Docking	2017	[43]
20	TKI.17	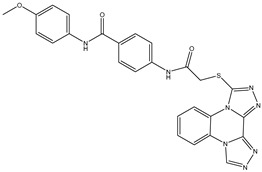	VEGFR-2	3200	ADMET /Docking	2021	[44]
21	TKI.18	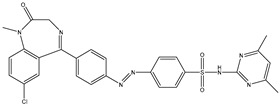	VEGFR-2	100	Docking	2020	[45]
22	TKI.19	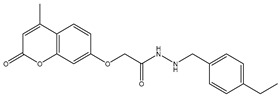	VEGFR-2	360	ADMET /Docking	2020	[46]
23	TKI.20a	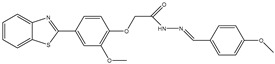	VEGFR-2/FGFR-1/PDGFR-β	190	ADMET /Docking	2020	[47]
24	TKI.20b	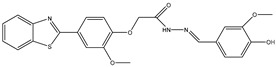	VEGFR-2/FGFR-1/PDGFR-β	170	ADMET /Docking	2020	
25	TKI.21a	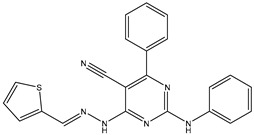	EGFR	373	Docking	2020	[48]
26	TKI.21b	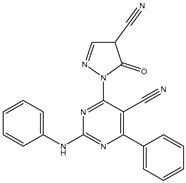	EGFR	369	Docking	2020	
27	AIK.1	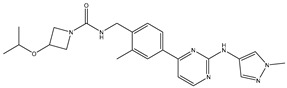	BTK	1	Docking	2020	[49]
28	AIK.3	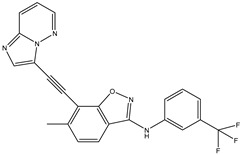	DDR1	13	Docking	2019	[50]
29	DDK.8	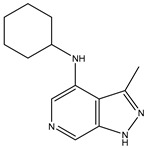	LRRK2	770	Docking	2019	[51]

**Table 3:** An illustration of individual and mean value calculation of the molecular descriptors for TKI.1.

ΤΚΙ.1	Molinspiration	Molsoft	SwissADME	Mcule	AdmetLab	pkSCM	Deep-PK	admetSAR	MEAN
Molecular weight (MW— gr/mole)	329.33	329.12	329.33	329.32	329.12	329.33	329.33	329.33	329.28
Total Polar Surface Area (TPSA—Å^2^)	76.51	59.73	76.50	76.50	76.50	nd *	76.50	76.50	74.11
Molar Refractivity (MR)	nd	nd	89.01	89.01	nd	nd	nd	nd	89.01
Log P_o/w_	3.73	2.61	3.11	3.62	2.54	3.54	2.41	2.67	3.03
Num. rotatable bonds (nRbs)	4	nd	4	4	4	4	4	4	4
Num. Hbond acceptors (nHAc)	6	5	6	7	6	6	6	6	6
Num. Hbond donors (nHDr)	2	2	2	2	2	2	2	2	2
Num. Rings (nRing)	nd	nd	nd	3	3	nd	nd	nd	3

* nd = not determined by specific platform.

**Table 4:** An illustration of ADME descriptors and the authors’ comprehensive evaluation for TKI.18.

ΤΚΙ.18	PreADMET	SwissADME	AdmetLab 3.0	pkSCM	Deep-PK	admetSAR 3.0	Authors’ Assessment of Overall Evidence
Bioavailability							
Caco-2 Permeability (LogPapp)	moderate	nd *	high	moderate	moderate	moderate	moderate
Human Intestinal Absorption	high	low	high	high	high	high	high
MDCK Permeability (Papp)	low	nd	moderate	nd	high	low	low
Pgp-substrate	nd	No	nd	Yes	No	No	No
Pgp-inhibitor	Yes	nd	nd	Yes	No	Yes	Yes
Distribution							
Plasma Protein Binding (PPB)	high	nd	high	nd	high	high	high
Excretion							
Total Clearance	nd	nd	low	moderate	low	low	low

* nd= not determined by specific platform.

**Table 5:** An illustration of toxicity and authors’ comprehensive evaluation for **TKI.20a**.

ΤΚΙ.20a	PreADMET	T.E.S.T	AdmetLab 3.0	pkSCM	Deep-PK	admetSAR 3.0	Authors’ Assessment of Overall Evidence
Carcinogenic potential							
Mutagenicity (Ames test)	positive	positive	positive	negative	positive	positive	positive
Carcinogenicity (rat)	negative	nd *	positive	nd	negative	negative	negative
Organ toxicity							
hERG Blockers	active	nd	inactive	inactive	inactive	inactive	inactive
Hepatotoxicity	nd	nd	positive	positive	positive	positive	positive

* nd = not determined by specific platform.

**Table 6:** Druglikeness evaluation according to Lipinski, CMC-like, Veber, Egan, Muegge, MMDR-like, and QED rules of FDA-approved tyrosine kinase inhibitors.

ID	Druglikeness							
	Lipinski Rule	Ghose/CMC-like Rule	Veber Rule	Egan Rule	Muegge Rule	MMDR-like Rules	QED	Score ^1^
Filgotinib	pass	pass	pass	pass	pass	pass	0.671	6.671
Sunitinib	pass	pass	pass	pass	pass	pass	0.626	6.626
Fruquintinib	pass	pass	pass	pass	pass	pass	0.55	6.550
Lenvatinib	pass	pass	pass	pass	pass	pass	0.549	6.549
Vandetanib	pass	pass	pass	pass	pass	pass	0.542	6.542
Axitinib	pass	pass	pass	pass	pass	pass	0.524	6.524
Gefitinib	pass	pass	pass	pass	pass	pass	0.518	6.518
Asciminib	pass	pass	pass	pass	pass	pass	0.498	6.498
Capivasertib	pass	pass	pass	pass	pass	pass	0.477	6.477
Pexidartinib	pass	pass	pass	pass	pass	pass	0.466	6.466
Pirtobrutinib	pass	pass	pass	pass	pass	pass	0.448	6.448
Erlotinib	pass	pass	pass	pass	pass	pass	0.418	6.418
Tivozanib	pass	pass	pass	pass	pass	pass	0.388	6.388
Momelotinib	pass	pass	pass	pass	pass	pass	0.598	6.598
Tofacitinib	pass	pass	pass	pass	pass	mid-structure	0.928	5.928
Ritlecitinib	pass	pass	pass	pass	pass	mid-structure	0.845	5.845
Abrocitinib	pass	pass	pass	pass	pass	mid-structure	0.835	5.835
Ruxolitinib	pass	pass	pass	pass	pass	mid-structure	0.8	5.800
Upadacitinib	pass	pass	pass	pass	pass	mid-structure	0.733	5.733
Baricitinib	pass	pass	pass	pass	pass	mid-structure	0.717	5.717
Larotrectinib	pass	pass	pass	pass	pass	mid-structure	0.67	5.670
Repotrectinib	pass	pass	pass	pass	pass	mid-structure	0.648	5.648
Lorlatinib	pass	pass	pass	pass	pass	mid-structure	0.615	5.615
Pemigatinib	pass	fail	pass	pass	pass	pass	0.572	5.572
Crizotinib	pass	pass	pass	pass	pass	mid-structure	0.533	5.533
Zanubrutinib	pass	fail	pass	pass	pass	pass	0.524	5.524
Futibatinib	pass	pass	pass	pass	pass	mid-structure	0.508	5.508
Deucravacitinib	pass	pass	pass	fail	pass	pass	0.496	5.496
Pazopanib	pass	pass	pass	pass	pass	mid-structure	0.492	5.492
Capmatinib	pass	pass	pass	pass	pass	mid-structure	0.489	5.489
Ibrutinib	pass	fail	pass	pass	pass	pass	0.467	5.467
Dasatinib	pass	fail	pass	pass	pass	pass	0.466	5.466
Dacomitinib	pass	fail	pass	pass	pass	pass	0.465	5.465
Afatinib	pass	fail	pass	pass	pass	pass	0.457	5.457
Erdafitinib	pass	fail	pass	pass	pass	pass	0.413	5.413
Regorafenib	pass	fail	pass	pass	pass	pass	0.407	5.407
Tepotinib	pass	fail	pass	pass	pass	pass	0.385	5.385
Tucatinib	pass	fail	pass	pass	pass	pass	0.358	5.358
Gilteritinib	1 violation	fail	pass	pass	pass	pass	0.428	4.928
Bosutinib	1 violation	fail	pass	pass	pass	pass	0.379	4.879
Selpercatinib	1 violation	fail	pass	pass	pass	pass	0.37	4.870
Brigatinib	1 violation	fail	pass	pass	pass	pass	0.352	4.852
Nintedanib	1 violation	fail	pass	pass	pass	pass	0.35	4.850
Cabozantinib	1 violation	fail	pass	pass	pass	pass	0.308	4.808
Pralsetinib	1 violation	fail	pass	pass	pass	pass	0.307	4.807
Pacritinib	pass	fail	pass	pass	pass	mid-structure	0.538	4.538
Acalabrutinib	pass	fail	pass	pass	pass	mid-structure	0.447	4.447
Avapritinib	pass	fail	pass	pass	pass	mid-structure	0.394	4.394
Osimertinib	pass	fail	fail	pass	pass	pass	0.311	4.311
Ponatinib	1 violation	fail	pass	pass	pass	mid-structure	0.394	3.894
Neratinib	1 violation	fail	fail	pass	pass	pass	0.218	3.718
Mobocertinib	1 violation	fail	fail	pass	pass	pass	0.174	3.674
Infigratinib	fail	fail	pass	pass	fail	pass	0.381	3.381
Ripretinib	fail	fail	pass	pass	fail	pass	0.323	3.323
Entrectinib	fail	fail	pass	pass	fail	pass	0.294	3.294
Ceritinib	fail	fail	pass	pass	fail	pass	0.279	3.279
Nilotinib	fail	fail	pass	pass	fail	pass	0.266	3.266
Alectinib	1 violation	fail	pass	pass	fail	mid-structure	0.582	3.082
Midostaurin	1 violation	fail	pass	pass	fail	mid-structure	0.287	2.787
Fedratinib	fail	fail	fail	pass	fail	pass	0.346	2.346
Quizatinib	fail	fail	pass	fail	fail	pass	0.257	2.257
Lapatinib	fail	fail	fail	pass	fail	pass	0.179	2.179
Fostamatinib	fail	fail	fail	fail	fail	pass	0.256	1.256

^1^ Scoring index: Pass (green) = 1, fail (red) = 0, 1 violation (yellow) = 0.5, mid-structure (blue) = 0, minimum acceptable score = 3.501.

**Table 7:** Criteria for the accomplishment of Medicinal Chemistry (Leadlikeness, GSK, PAINS, Brenk) rules in FDA-approved tyrosine kinase inhibitors.

ID	Medicinal Chemistry				
	Leadlikeness	GSK Rule	PAINS (SwissADME)	Brenk (SwissADME)	Score ^1^
Abrocitinib	pass	pass	pass	pass	4
Ruxolitinib	pass	pass	pass	pass	4
Tofacitinib	pass	pass	pass	pass	4
Baricitinib	fail	pass	pass	pass	3
Fruquintinib	fail	pass	pass	pass	3
Repotrectinib	fail	pass	pass	pass	3
Ritlecitinib	pass	pass	pass	fail	3
Upadacitinib	fail	pass	pass	pass	3
Alectinib	fail	fail	pass	pass	2
Avapritinib	fail	fail	pass	pass	2
Axitinib	fail	fail	pass	pass	2
Bosutinib	fail	fail	pass	pass	2
Capivasertib	fail	fail	pass	pass	2
Capmatinib	fail	fail	pass	pass	2
Ceritinib	fail	fail	pass	pass	2
Dasatinib	fail	fail	pass	pass	2
Deucravacitinib	fail	fail	pass	pass	2
Entrectinib	fail	fail	pass	pass	2
Erdafitinib	fail	fail	pass	pass	2
Erlotinib	fail	pass	pass	fail	2
Fedratinib	fail	fail	pass	pass	2
Filgotinib	fail	fail	pass	pass	2
Gefitinib	fail	fail	pass	pass	2
Lapatinib	fail	fail	pass	pass	2
Larotrectinib	fail	fail	pass	pass	2
Lenvatinib	fail	fail	pass	pass	2
Lorlatinib	fail	fail	pass	pass	2
Midostaurin	fail	fail	pass	pass	2
Nilotinib	fail	fail	pass	pass	2
Pazopanib	fail	fail	pass	pass	2
Pemigatinib	fail	fail	pass	pass	2
Pexidartinib	fail	fail	pass	pass	2
Pirtobrutinib	fail	fail	pass	pass	2
Pralsetinib	fail	fail	pass	pass	2
Quizatinib	fail	fail	pass	pass	2
Regorafenib	fail	fail	pass	pass	2
Ripretinib	fail	fail	pass	pass	2
Selpercatinib	fail	fail	pass	pass	2
Sunitinib	fail	pass	pass	fail	2
Tepotinib	fail	fail	pass	pass	2
Tivozanib	fail	fail	pass	pass	2
Tucatinib	fail	fail	pass	pass	2
Vandetanib	fail	fail	pass	pass	2
Acalabrutinib	fail	fail	pass	fail	1
Afatinib	fail	fail	pass	fail	1
Asciminib	fail	fail	pass	fail	1
Brigatinib	fail	fail	pass	fail	1
Cabozantinib	fail	fail	pass	fail	1
Crizotinib	fail	fail	pass	fail	1
Dacomitinib	fail	fail	pass	fail	1
Fostamatinib	fail	fail	pass	fail	1
Futibatinib	fail	fail	pass	fail	1
Gilteritinib	fail	fail	fail	pass	1
Ibrutinib	fail	fail	pass	fail	1
Infigratinib	fail	fail	fail	pass	1
Mobocertinib	fail	fail	pass	fail	1
Neratinib	fail	fail	pass	fail	1
Nintedanib	fail	fail	pass	fail	1
Osimertinib	fail	fail	pass	fail	1
Pacritinib	fail	fail	pass	fail	1
Ponatinib	fail	fail	pass	fail	1
Zanubrutinib	fail	fail	pass	fail	1
Momelotinib	fail	fail	fail	fail	0

^1^ Scoring index: Pass (green) = 1, fail (red) = 0, minimum acceptable score = 2.

**Table 8:** Bioavailability evaluation (Caco-2 Permeability, HIA, MDCK prermeability, Pgp-substrate/inhibitor) of FDA-approved tyrosine kinase inhibitors.

ID	Bioavailability						
	Caco-2 Permeability	Human Intestinal Absorption (HIA)	MDCK Permeability	Pgp-Substrate	Pgp- Inhibitor	Scoring ^1^	Authors’ Assessment of Overall Evidence
**Lorlatinib**	high	high	high	no	no	5	high
**Ruxolitinib**	high	high	high	no	no	5	high
**Baricitinib**	high	high	moderate	no	no	4.5	high
**Ritlecitinib**	high	high	moderate	no	no	4.5	high
**Abrocitinib**	moderate	high	moderate	no	no	4	high
**Capmatinib**	high	high	high	yes	yes	4	high
**Deucravacitinib**	moderate	high	moderate	no	no	4	high
**Filgotinib**	moderate	high	moderate	no	no	4	high
**Fruquintinib**	high	high	high	no	yes	4	high
**Futibatinib**	high	high	high	no	yes	4	high
**Gefitinib**	high	high	high	yes	yes	4	high
**Pemigatinib**	high	high	high	yes	yes	4	high
**Pexidartinib**	high	high	high	yes	yes	4	high
**Tofacitinib**	moderate	high	moderate	no	no	4	high
**Vandetanib**	high	high	high	yes	yes	4	high
**Cabozantinib**	moderate	high	high	yes	yes	3.5	high
**Erlotinib**	high	high	moderate	no	yes	3.5	high
**Fostamatinib**	moderate	high	low	no	no	3.5	high
**Infigratinib**	moderate	high	high	yes	yes	3.5	high
**Larotrectinib**	high	high	moderate	yes	yes	3.5	high
**Momelotinib**	high	high	moderate	yes	yes	3.5	high
**Pirtobrutinib**	moderate	high	high	yes	yes	3.5	high
**Ripretinib**	moderate	high	high	no	yes	3.5	high
**Tepotinib**	moderate	high	high	yes	yes	3.5	high
**Tivozanib**	moderate	high	high	no	yes	3.5	high
**Acalabrutinib**	high	high	low	yes	yes	3	high
**Axitinib**	high	high	low	yes	yes	3	high
**Brigatinib**	moderate	high	moderate	yes	yes	3	high
**Ceritinib**	moderate	high	moderate	yes	yes	3	high
**Crizotinib**	high	high	low	yes	yes	3	high
Dacomitinib	high	high	low	yes	yes	3	high
Fedratinib	moderate	high	moderate	yes	yes	3	high
Nilotinib	moderate	high	moderate	yes	yes	3	high
Pacritinib	high	high	low	yes	yes	3	high
Ponatinib	moderate	high	moderate	yes	yes	3	high
Pralsetinib	moderate	high	moderate	yes	yes	3	high
Quizatinib	moderate	high	moderate	yes	yes	3	high
Sunitinib	moderate	high	moderate	yes	yes	3	high
Tucatinib	high	high	low	yes	yes	3	high
Afatinib	moderate	high	low	yes	yes	2.5	low
Alectinib	moderate	high	low	yes	yes	2.5	low
Asciminib	moderate	high	low	no	yes	2.5	low
Avapritinib	moderate	high	low	yes	yes	2.5	low
Bosutinib	moderate	high	low	yes	yes	2.5	low
Dasatinib	moderate	high	low	yes	yes	2.5	low
Entrectinib	moderate	high	low	yes	yes	2.5	low
Erdafitinib	moderate	high	low	yes	yes	2.5	low
Gilteritinib	moderate	high	low	yes	yes	2.5	low
Ibrutinib	moderate	high	low	no	yes	2.5	low
Lapatinib	moderate	high	low	yes	yes	2.5	low
Midostaurin	moderate	high	low	yes	yes	2.5	low
Mobocertinib	moderate	high	low	yes	yes	2.5	low
Neratinib	moderate	high	low	yes	yes	2.5	low
Nintedanib	moderate	high	low	yes	yes	2.5	low
Osimertinib	moderate	high	low	yes	yes	2.5	low
Pazopanib	moderate	high	low	no	yes	2.5	low
Regorafenib	moderate	high	low	no	yes	2.5	low
Selpercatinib	moderate	high	low	yes	yes	2.5	low
Upadacitinib	moderate	high	high	yes	no	2.5	low
Zanubrutinib	moderate	high	low	yes	yes	2.5	low
Capivasertib	moderate	high	moderate	yes	no	2	low
Lenvatinib	moderate	high	moderate	yes	no	2	low
Repotrectinib	high	high	low	yes	no	2	low

^1^ Scoring index: high (green) = 1, moderate (yellow) = 0.5, low (red) = 0, No/No (green/red) = 2, No/Yes (green/green) = 1, Yes/Yes (red/green) = 1, Yes/No (red/red) = 0, minimum acceptable score = 2.51.

**Table 9:** Distribution (PPB) and Excretion (Total Clearance) evaluation of FDA-approved tyrosine kinase inhibitors.

ID	Distribution	ID	Excretion
	Plasma Protein Binding (PPB) ^1^		Total Clearance
Abrocitinib	low	Abrocitinib	high
Avapritinib	low	Acalabrutinib	low
Baricitinib	low	Afatinib	low
Brigatinib	low	Alectinib	high
Capivasertib	low	Asciminib	low
Crizotinib	low	Avapritinib	high
Deucravacitinib	low	Axitinib	low
Erdafitinib	low	Baricitinib	high
Filgotinib	low	Bosutinib	low
Fruquintinib	low	Brigatinib	low
Futibatinib	low	Cabozantinib	low
Gefitinib	low	Capmatinib	low
Gilteritinib	low	Capivasertib	low
Larotrectinib	low	Ceritinib	low
Mobocertinib	low	Crizotinib	low
Nintedanib	low	Dacomitinib	high
Pacritinib	low	Dasatinib	low
Pemigatinib	low	Deucravacitinib	low
Repotrectinib	low	Entrectinib	low
Ritlecitinib	low	Erdafitinib	low
Ruxolitinib	low	Erlotinib	low
Sunitinib	low	Fedratinib	low
Tofacitinib	low	Filgotinib	low
Upadacitinib	low	Fostamatinib	low
Vandetanib	low	Fruquintinib	low
Zanubrutinib	low	Futibatinib	low
Acalabrutinib	high	Gefitinib	low
Afatinib	high	Gilteritinib	high
Alectinib	high	Ibrutinib	low
Asciminib	high	Infigratinib	low
Axitinib	high	Lapatinib	low
Bosutinib	high	Larotrectinib	low
Cabozantinib	high	Lenvatinib	low
Capmatinib	high	Lorlatinib	low
Ceritinib	high	Midostaurin	low
Dacomitinib	high	Mobocertinib	low
Dasatinib	high	Momelotinib	low
Entrectinib	high	Neratinib	low
Erlotinib	high	Nilotinib	low
Fedratinib	high	Nintedanib	low
Fostamatinib	high	Osimertinib	low
Ibrutinib	high	Pacritinib	low
Infigratinib	high	Pazopanib	low
Lapatinib	high	Pemigatinib	high
Lenvatinib	high	Pexidartinib	high
Lorlatinib	high	Pirtobrutinib	low
Midostaurin	high	Ponatinib	low
Momelotinib	high	Pralsetinib	low
Neratinib	high	Quizatinib	low
Nilotinib	high	Regorafenib	low
Osimertinib	high	Repotrectinib	low
Pazopanib	high	Ripretinib	low
Pexidartinib	high	Ritlecitinib	low
Pirtobrutinib	high	Ruxolitinib	high
Ponatinib	high	Selpercatinib	low
Pralsetinib	high	Sunitinib	high
Quizatinib	high	Tepotinib	high
Regorafenib	high	Tivozanib	low
Ripretinib	high	Tofacitinib	high
Selpercatinib	high	Tucatinib	low
Tepotinib	high	Upadacitinib	low
Tivozanib	high	Vandetanib	high
Tucatinib	high	Zanubrutinib	low

^1^ Color index: high = red, low = green, acceptable color = green.

**Table 10:** Toxicity evaluation (carcinogenic potential and organ toxicity) of FDA-approved tyrosine kinase inhibitors.

ID	Toxicity				
	Carcinogenic Potential		Organ Toxicity		Score ^1^
	Ames Test	Carcinogencity (Rat)	hERG Blockers	Hepatotoxicity	
Baricitinib	negative	negative	inactive	negative	4
Brigatinib	negative	negative	inactive	negative	4
Dasatinib	negative	negative	inactive	negative	4
Tofacitinib	negative	negative	inactive	negative	4
Abrocitinib	negative	negative	inactive	positive	3
Bosutinib	negative	negative	active	negative	3
Capivasertib	negative	negative	active	negative	3
Dacomitinib	negative	negative	active	negative	3
Deucravacitinib	negative	negative	inactive	positive	3
Entrectinib	negative	negative	active	negative	3
Filgotinib	negative	positive	inactive	negative	3
Fostamatinib	negative	negative	inactive	positive	3
Neratinib	negative	negative	active	negative	3
Nilotinib	negative	negative	active	negative	3
Pemigatinib	negative	positive	inactive	negative	3
Ponatinib	negative	negative	active	negative	3
Regorafenib	negative	negative	inactive	positive	3
Ruxolitinib	negative	positive	inactive	negative	3
Selpercatinib	positive	negative	inactive	negative	3
Tepotinib	negative	negative	active	negative	3
Upadacitinib	negative	positive	inactive	negative	3
Acalabrutinib	positive	negative	active	negative	2
Alectinib	negative	positive	active	negative	2
Avapritinib	negative	positive	active	negative	2
Axitinib	positive	negative	inactive	positive	2
Ceritinib	negative	negative	active	positive	2
Crizotinib	positive	negative	active	negative	2
Erdafitinib	negative	positive	active	negative	2
Fedratinib	negative	negative	active	positive	2
Futibatinib	positive	negative	active	negative	2
Gefitinib	positive	negative	active	negative	2
Infigratinib	negative	negative	active	positive	2
Lapatinib	positive	negative	inactive	positive	2
Lorlatinib	positive	positive	inactive	negative	2
Midostaurin	negative	positive	active	negative	2
Nintedanib	negative	negative	active	positive	2
Pazopanib	negative	positive	inactive	positive	2
Pexidartinib	negative	negative	active	positive	2
Quizatinib	negative	positive	active	negative	2
Vandetanib	negative	positive	active	negative	2
Zanubrutinib	positive	negative	active	negative	2
Afatinib	positive	negative	active	positive	1
Asciminib	positive	positive	active	negative	1
Capmatinib	positive	positive	active	negative	1
Erlotinib	positive	positive	active	negative	1
Fruquintinib	positive	positive	inactive	positive	1
Gilteritinib	positive	positive	active	negative	1
Larotrectinib	positive	positive	inactive	positive	1
Lenvatinib	positive	positive	active	negative	1
Mobocertinib	negative	positive	active	positive	1
Momelotinib	positive	positive	inactive	positive	1
Pacritinib	negative	positive	active	positive	1
Pirtobrutinib	positive	positive	active	negative	1
Pralsetinib	negative	positive	active	positive	1
Ripretinib	positive	positive	inactive	positive	1
Ritlecitinib	positive	positive	inactive	positive	1
Tivozanib	negative	positive	active	positive	1
Tucatinib	negative	positive	active	positive	1
Cabozantinib	negative	positive	active	positive	1
Ibrutinib	positive	positive	active	positive	0
Osimertinib	positive	positive	active	positive	0
Repotrectinib	positive	positive	active	positive	0
Sunitinib	positive	positive	active	positive	0

^1^ Scoring index: Positive (red) = 0, negative (green) = 1, active (red) = 0, inactive (green) = 1, minimum acceptable score = 2.01.

**Table 11:** DrugBank calculated, experimental, and average predicted MW, TPSA, MR, and LogP_o/w_ values.

Drug	Physicochemical Properties								
	Molecular Weight (g/mol)		TPSA (Å^2^)		Molar Refractivity		Log P_o/w_		
	Calculated DrugBank (yi)	Predicted ($\hat{yi}$)	Calculated DrugBank (yi)	Predicted ($\hat{yi}$)	Calculated DrugBank (yi)	Predicted ($\hat{yi}$)	Experi-mental (yi)	Predicted ($\hat{yi}$)	Molinspi-Ration/Molsoft ($\hat{yi}$)
Abrocitinib	323.42	323.35	90.98	91.35	86.00	86.59	*	1.56	-
Acalabrutinib	465.52	465.43	118.51	114.06	135.72	136.51	0.49	3.04	2.50
Afatinib	485.94	485.75	88.61	86.09	131.38	129.90	*	3.95	-
Alectinib	482.62	482.54	72.36	69.80	155.11	149.50	*	5.00	-
Asciminib	449.84	449.66	103.37	100.27	104.81	113.26	*	3.20	-
Avapritinib	498.57	498.48	106.29	102.86	164.55	144.37	*	2.58	-
Axitinib	386.47	386.39	70.67	76.06	115.14	112.82	*	4.04	-
Baricitinib	371.42	371.35	120.56	119.33	105.55	98.51	*	0.63	-
Bosutinib	530.45	530.08	82.88	80.03	142.12	150.65	3.34	4.59	4.55
Brigatinib	584.10	583.89	85.86	86.30	164.77	176.58	5.17	4.08	4.53
Cabozantinib	501.51	501.42	98.78	95.78	136.12	136.59	*	4.49	-
Capivasertib	428.92	428.73	120.16	114.08	116.97	118.97	*	1.67	-
Capmatinib	412.43	412.35	85.07	81.96	125.27	113.93	*	2.94	-
Ceritinib	558.14	557.91	105.24	104.90	153.86	158.71	*	5.62	-
Crizotinib	450.34	450.04	77.99	75.79	128.43	120.72	1.83	4.27	3.43
Dacomitinib	469.94	469.75	79.38	77.06	129.91	132.73	3.92	4.96	4.90
Dasatinib	488.01	487.80	106.51	111.29	133.08	138.63	1.80	3.39	3.50
Deucravacitinib	425.47	424.64	135.95	132.40	138.38	113.15	*	1.82	-
Entrectinib	560.65	560.54	85.52	83.34	161.24	163.44	*	4.88	-
Erdafitinib	446.56	446.47	77.33	74.91	139.32	131.48	*	4.00	-
Erlotinib	393.44	393.37	74.73	72.77	107.79	111.40	2.70	2.93	2.50
Fedratinib	524.68	524.58	108.48	108.45	147.88	151.66	*	4.97	-
Filgotinib	425.51	425.42	96.67	96.15	126.12	118.09	*	1.98	-
Fostamatinib	580.46	580.39	186.72	183.88	137.1	141.80	*	2.30	-
Fruquintinib	393.40	393.33	95.71	92.80	106.25	106.77	*	2.92	-
Futibatinib	418.46	418.39	108.39	104.89	122.82	119.66	*	2.22	-
Gefitinib	446.90	446.72	68.74	66.93	117.51	121.66	3.20	4.06	3.82
Gilteritinib	552.72	552.63	121.11	117.42	159.84	168.43	4.35	2.67	3.04
Ibrutinib	440.51	440.43	99.16	95.66	138.07	131.01	3.97	3.85	3.49
Infigratinib	560.48	560.16	95.09	92.08	152.71	159.27	*	4.85	-
Lapatinib	581.06	580.83	106.35	105.71	152.42	153.88	5.40	5.67	5.64
Larotrectinib	428.44	428.38	86	82.90	122.96	117.01	*	2.57	-
Lenvatinib	426.86	426.67	115.57	111.99	112.21	112.86	3.30	3.24	3.14
Lorlatinib	406.42	406.35	110.06	106.65	121.17	111.44	*	2.15	-
Midostaurin	570.65	571.79	77.73	74.48	162.61	169.20	5.89	4.74	4.66
Mobocertinib	585.71	585.61	113.85	110.30	171.52	171.32	*	4.52	-
Momelotinib	414.47	414.39	103.17	100.20	118.46	120.29	*	2.61	-
Neratinib	557.05	556.84	112.4	108.35	157.29	157.05	*	4.80	-
Nilotinib	529.52	529.44	97.62	94.10	152.85	141.08	5.01	5.33	5.14
Nintedanib	539.62	539.54	94.22	92.56	159.1	167.00	3.00	3.17	3.5
Osimertinib	499.62	499.53	87.55	84.72	150.32	150.43	*	3.92	-
Pacritinib	472.59	472.50	68.74	67.92	139.43	143.91	*	4.29	-
Pazopanib	437.52	437.43	119.03	118.07	132.18	121.50	*	3.29	-
Pemigatinib	487.51	487.43	83.16	80.71	125.32	136.22	*	2.99	-
Pexidartinib	417.82	417.64	66.49	63.64	105.89	104.94	*	4.31	-
Pirtobrutinib	479.44	479.36	125.26	121.68	127.89	115.16	*	3.22	-
Ponatinib	532.56	532.48	65.77	63.49	152.63	150.10	*	4.45	-
Pralsetinib	533.61	533.52	135.53	131.05	146.12	143.26	*	3.52	-
Quizatinib	560.67	560.57	106.16	110.87	168.24	160.37	*	5.70	-
Regorafenib	482.82	482.63	92.35	89.51	114.73	112.44	*	5.06	-
Repotrectinib	355.37	355.30	80.55	78.61	106.42	100.58	*	2.28	-
Ripretinib	510.37	510.04	86.36	85.05	133.78	133.19	5.63	4.95	5.29
Ritlecitinib	285.35	285.30	73.91	71.52	82.84	86.07	*	1.52	-
Ruxolitinib	306.37	306.32	83.18	80.38	98.01	87.66	*	2.41	-
Selpercatinib	525.61	525.52	112.04	107.85	158.75	152.98	*	3.35	-
Sunitinib	398.47	398.41	77.23	75.97	116.27	116.31	*	3.00	-
Tepotinib	492.58	492.53	94.71	93.92	154.74	145.45	*	3.95	-
Tivozanib	454.86	454.68	107.74	104.42	120.85	120.00	4.31	4.60	4.28
Tofacitinib	312.37	312.32	88.91	85.66	87.8	91.20	1.81	1.03	0.65
Tucatinib	480.53	480.45	110.85	106.83	148.37	141.66	3.62	4.52	4.46
Upadacitinib	380.38	380.32	78.32	74.97	93.03	96.54	*	2.50	-
Vandetanib	475.35	475.05	59.51	57.76	118.63	123.26	5.00	4.63	4.67
Zanubrutinib	471.56	471.48	102.48	99.64	146.25	141.35	*	3.49	-
Average ($\bar{y}$)	467.46	-	96.66	-	132.43	-	3.69	-	-

* Experimental data for these values were not found; - predicted data for these values were not calculated.

**Table 12:** Regression coefficients table for MW, TPSA, and MR.

**Molecular Weight**									
**Model**		**Coefficients**			**Evaluation Metrics**			**95.0% Confidence Interval for B**	
		B	r	R^2^	*RMSE*	*MAE*	*RMSE_baseline_*	Lower Bound	Upper Bound
1	(Constant)	0.159	-	-	-	-	-	−0.172	0.489
	MW predicted	1.000	1.000	1.000	0.230	0.152	72.469	0.999	1.001
Dependent Variable: MWdrugbank									
**y = 1.000 (±0.001) × x + 0.159 (±0.331)**									
**TPSA**									
1	(Constant)	1.057	-	-	-	-	-	−1.333	3.447
	TPSA predicted	1.012	0.995	0.991	2.995	2.712	21.090	0.988	1.037
Dependent Variable: TPSAdrugbank									
**y = 1.012 (±0.024) × x + 1.057 (±2.190)**									
**Molar Refractivity**									
1	(Constant)	12.326	-	-	-	-	-	2.058	22.593
	MR predicted	0.917	0.950	0.902	7.249	5.521	21.919	0.840	0.994
Dependent Variable: MRdrugbank									
**y = 0.917 (±0.077) × x + 12.326 (±10.268)**									

**Table 13:** Regression coefficients table for LogP_o/w_.

**Log P_o/w_**									
**Model**		**Coefficients**			**Evaluation Metrics**			**95.0% Confidence Interval for B**	
		B	r	R^2^	*RMSE*	*MAE*	*RMSE_baseline_*	Lower Bound	Upper Bound
1	(Constant)	2.213	-	-	-	-	-	1.098	3.327
	Experimental	0.481	0.645	0.417	1.142	0.892	1.430	0.199	0.763
Dependent Variable: Log P_o/w_ Predicted									
**y = 0.481 (±0.282) × x + 2.213 (±1.115)**									
**Log P_o/w_** (**Molinspiration–Molsoft)**									
1	(Constant)	1.658	-	-	-	-	-	0.615	2.702
	Experimental	0.603	0.750	0.562	0.970	0.786	1.430	0.339	0.867
Dependent Variable: Molinspiration–Molsoft									
**y = 0.603 (±0.264) × x + 1.658 (±1.043)**									

**Table 14:** Experimental and predicted Bioavailability, PPB, Clearance and Toxicity (Ames test, Carcinogenicity, hERG Blockers and Hepatotoxicity) data.

Drug	Pharmacokinetic Properties						Toxicity							
	Bioavail-Ability ^1^		Plasma Protein Binding (PPB) ^1^		Clearance (L/h) ^1^		Ames Test ^1^		Carcinogenicity ^1^		hERG Blockers (Human Ether-à-go-go-Related Gene) ^1^		Hepatotoxicity ^1^	
	Exp	Pred	Exp	Pred	Exp	Pred	Exp	Pred	Exp	Pred	Exp	Pred	Exp	Pred
Abrocitinib	high	high	low	low	*	high	negative	negative	negative	negative	*	negative	negative	positive
Acalabrutinib	low	high	high	high	high	low	*	positive	negative	negative	*	positive	positive	negative
Afatinib	high	low	high	high	high	low	*	positive	*	negative	*	positive	positive	positive
Alectinib	low	low	high	high	high	high	negative	negative	*	positive	*	positive	positive	negative
Asciminib	*	low	high	high	low	low	negative	positive	*	positive	*	positive	negative	negative
Avapritinib	high	low	high	low	low	high	negative	negative	*	positive	*	positive	negative	negative
Axitinib	high	high	high	high	low	low	positive	positive	*	negative	negative	negative	positive	positive
Baricitinib	high	high	low	low	low	high	negative	negative	negative	negative	*	negative	negative	negative
Bosutinib	low	low	high	high	high	low	negative	negative	negative	negative	negative	positive	positive	negative
Brigatinib	low	high	low	low	low	low	negative	negative	*	negative	*	negative	positive	negative
Cabozantinib	high	high	high	high	low	low	negative	negative	negative	positive	*	positive	positive	positive
Capivasertib	low	low	low	low	low	low	*	negative	negative	negative	*	positive	negative	negative
Capmatinib	high	high	high	high	low	low	*	positive	negative	positive	*	positive	positive	negative
Ceritinib	*	high	high	high	low	low	negative	negative	*	negative	*	positive	positive	positive
Crizotinib	low	high	high	low	high	low	negative	positive	*	negative	positive	positive	positive	negative
Dacomitinib	high	high	high	high	low	high	negative	negative	*	negative	*	positive	negative	negative
Dasatinib	high	low	high	high	high	low	negative	negative	positive	negative	negative	negative	positive	negative
Deucravacitinib	high	high	low	low	low	low	negative	negative	negative	negative	*	negative	negative	positive
Entrectinib	*	low	high	high	low	low	negative	negative	*	negative	*	positive	positive	negative
Erdafitinib	high	low	high	low	low	low	negative	negative	*	positive	*	positive	negative	negative
Erlotinib	high	high	high	high	low	low	negative	positive	positive	positive	negative	positive	positive	negative
Fedratinib	*	high	high	high	low	low	negative	negative	negative	negative	*	positive	positive	positive
Filgotinib	*	high	*	low	*	low	*	negative	*	positive	*	negative	*	negative
Fostamatinib	high	high	high	high	*	low	negative	negative	negative	negative	*	negative	positive	positive
Fruquintinib	*	high	high	low	low	low	negative	positive	*	positive	*	negative	positive	positive
Futibatinib	*	high	high	low	low	low	negative	positive	*	negative	*	positive	negative	negative
Gefitinib	high	high	low	low	*	low	negative	positive	positive	negative	positive	positive	positive	negative
Gilteritinib	*	low	high	low	low	high	negative	positive	*	positive	*	positive	negative	negative
Ibrutinib	low	low	high	high	high	low	negative	positive	negative	positive	*	positive	positive	positive
Infigratinib	*	high	high	high	low	low	negative	negative	*	negative	*	positive	negative	positive
Lapatinib	*	low	high	high	*	low	negative	positive	negative	negative	positive	negative	positive	positive
Larotrectinib	low	high	low	low	high	low	negative	positive	*	positive	*	negative	positive	positive
Lenvatinib	high	low	high	high	*	low	negative	positive	*	positive	*	positive	positive	negative
Lorlatinib	high	high	low	high	low	low	negative	positive	*	positive	*	negative	positive	negative
Midostaurin	*	low	high	high	*	low	negative	negative	*	positive	*	positive	negative	negative
Mobocertinib	low	low	high	low	high	low	negative	negative	*	positive	*	positive	negative	positive
Momelotinib	*	high	high	high	high	low	negative	positive	negative	positive	*	negative	positive	positive
Neratinib	high	low	high	high	high	low	negative	negative	negative	negative	*	positive	positive	negative
Nilotinib	low	high	high	high	low	low	negative	negative	negative	negative	positive	positive	positive	negative
Nintedanib	low	low	high	low	high	low	negative	negative	negative	negative	*	positive	positive	positive
Osimertinib	low	low	high	high	low	low	negative	positive	positive	positive	*	positive	*	positive
Pacritinib	*	high	high	low	low	low	negative	negative	negative	positive	*	positive	negative	positive
Pazopanib	low	low	high	high	*	low	negative	negative	positive	positive	negative	negative	positive	positive
Pemigatinib	low	high	high	low	low	high	negative	negative	*	positive	*	negative	negative	negative
Pexidartinib	*	high	high	high	low	high	negative	negative	negative	negative	*	positive	negative	positive
Pirtobrutinib	high	high	high	high	low	low	negative	positive	*	positive	*	positive	positive	negative
Ponatinib	*	high	high	high	*	low	negative	negative	positive	negative	*	positive	negative	negative
Pralsetinib	*	high	high	high	low	low	negative	negative	*	positive	*	positive	positive	positive
Quizatinib	*	high	*	high	*	low	*	negative	*	positive	*	positive	*	negative
Regorafenib	high	low	high	high	*	low	*	negative	*	negative	negative	negative	positive	positive
Repotrectinib	low	low	high	low	low	low	negative	positive	*	positive	*	positive	positive	positive
Ripretinib	high	high	high	high	low	low	negative	positive	*	positive	*	negative	negative	positive
Ritlecitinib	high	high	low	low	*	low	negative	positive	positive	positive	*	negative	negative	positive
Ruxolitinib	high	high	high	low	low	high	negative	negative	negative	positive	negative	negative	negative	negative
Selpercatinib	high	low	high	high	low	low	negative	positive	negative	negative	*	negative	positive	negative
Sunitinib	high	high	high	low	low	high	negative	positive	positive	positive	positive	positive	positive	positive
Tepotinib	high	high	high	high	low	high	*	negative	*	negative	*	positive	positive	negative
Tivozanib	high	high	high	high	low	low	*	negative	*	positive	*	positive	negative	positive
Tofacitinib	high	high	low	low	*	high	negative	negative	negative	negative	*	negative	negative	negative
Tucatinib	*	high	high	high	high	low	negative	negative	*	positive	*	positive	positive	positive
Upadacitinib	*	low	low	low	*	low	negative	negative	negative	positive	*	negative	negative	negative
Vandetanib	high	high	high	low	*	high	negative	negative	*	positive	positive	positive	negative	negative
Zanubrutinib	*	low	high	low	high	low	negative	positive	*	negative	*	positive	positive	negative

* Experimental data for these values were not found. **^1^** In this table, colors (red/green) are applied to enhance comparison.

**Table 15:** Compounds eliminated by the screening technique for each criterion, arranged by the total number of hits.

Top 24—Druglikeness		Top 16—Med. Chemistry	Top 21—Bioavailability	Top 2—Distribution	Top 9—Overall Toxicity
1	AIK.1	TKI.16	TKI.16	TKI.16	TKI.19
2	TKI.19	DDK8	DDK.8	AIK.1	DDK8
3	DDK8	TKI.19	TKI.19		TKI.4
4	TKI.21b	TKI.21b	TKI.21b		TKI.21b
5	TKI.16	AIK.1	TKI.4		TKI.8
6	TKI.4	TKI.4	AIK.1		TKI.20b
7	TKI.20b	TKI.14b	TKI.14a		TKI.18
8	TKI.1	TKI.8	TKI.14b		TKI.13b
9	TKI.14b	TKI.6	TKI.6		TKI.13a
10	TKI.14a	TKI.2a	TKI.1		
11	TKI.2b	TKI.2b	TKI.20b		
12	TKI.2a	TKI.14a	TKI.2a		
13	TKI.8	TKI.1	TKI.2b		
14	TKI.6	TKI.17	TKI.10		
15	TKI.20a	TKI.11	TKI.5		
16	TKI.10	TKI.15	TKI.21a		
17	TKI.5		TKI.9		
18	TKI.9		TKI.20a		
19	TKI.21a		AIK.3		
20	TKI.17		TKI.3		
21	TKI.18		TKI.7a		
22	TKI.13a				
23	TKI.13b				
24	TKI.11				

**Table 16:** Compounds identified through our novel screening approach, demonstrating the percentage of criteria met, points of violation, and reported biological targets.

D-ADMET Screening					
A/A	Compound	Structure	Criteria Meeting (≥60%)	Violation Points	Reported Biological Target
1	TKI.1	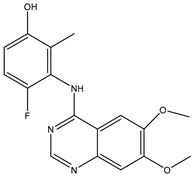	60	Distribution, Overall Toxicity	Ret
2	TKI.2a	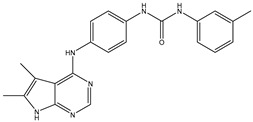	60	Distribution, Overall Toxicity	VEGFR-2
3	TKI.2b	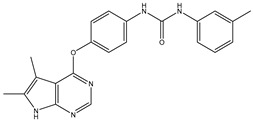	60	Distribution, Overall Toxicity	VEGFR-2
4	TKI.4	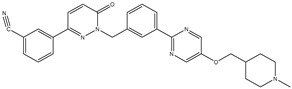	80	Distribution	c-Met
5	TKI.6	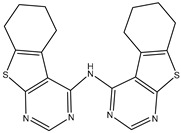	60	Distribution, Overall Toxicity	dual EGFR/HER2
6	TKI.8	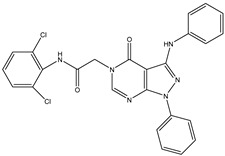	60	Distribution, Bioavailability	EGFR
7	TKI.14a	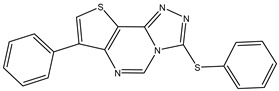	60	Distribution, Overall Toxicity	EGFR
8	TKI.14b	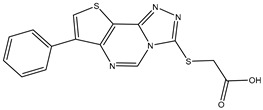	60	Distribution, Overall Toxicity	
9	TKI.16	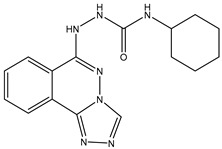	80	Overall Toxicity	VEGFR-2
10	TKI.19	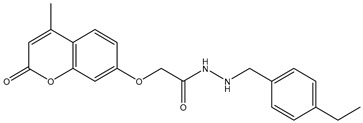	80	Distribution	VEGFR-2
11	TKI.20b	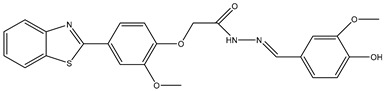	60	Medicinal Chemsistry, Distribution	VEGFR-2/FGFR-1/PDGFR-β
12	TKI.21b	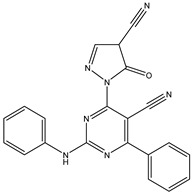	80	Distribution	EGFR
13	AIK.1	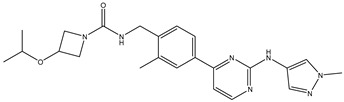	80	Overall Toxicity	BTK
14	DDK.8	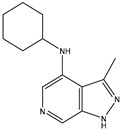	80	Distribution	LRRK2

**Table 17:** Results from cross-docking demonstrating affinity, CNN pose score, CNN affinity, and RMSD values for known drugs pertaining to each biological target.

Target	Drug	Affinity (kcal/mol)	CNN Pose Score	CNN Affinity	Cross-Docking RMSD (Å)
**VEGFR-2 (PDB ID:4ASE)**	Axitinib	−8.53	0.842	7.634	5.500
	Cabozatinib	−11.86	0.913	7.725	1.059
	Fruquitinib	−8.74	0.906	7.672	1.499
	Lenvatinib	−11.03	0.957	8.049	2.776
	Pazopanib	−8.69	0.856	7.407	3.730
	Regorafenib	−11.24	0.890	7.833	1.688
	Sorafenib	−11.25	0.882	7.588	2.536
	Sunitinib	−7.35	0.728	7.312	5.250
	Vandetanib	−10.42	0.814	8.062	1.514
**HER2 (PDB ID:7PCD)**	Afatinib	−7.61	0.925	7.381	2.906
	Capivasertib	−9.71	0.898	7.45	2.537
	Lapatinib	−9.98	0.858	7.609	2.022
	Neratinib	−7.51	0.780	7.875	2.432
	Tucatinib	−10.64	0.750	7.634	1.494
**EGFR (PDB ID:7T4I)**	Afatinib	−8.35	0.900	7.852	2.169
	Dacomitinib	−8.60	0.932	8.125	2.186
	Gefitinib	−7.93	0.983	7.986	1.862
	Osimertinib	−7.12	0.932	7.948	1.562

**Table 18:** *T*-test for equality of means (independent samples test).

Independent Samples Test							
Molecular Descriptors	t	df	Significance	Mean Difference	Std. Error Difference	95% Confidence Interval of the Difference	
			Two-Sided (*p*)			Lower	Upper
Molecular weight	−0.010	47.245	0.992	−0.22	21.457	−43.384	42.937
TPSA	0.962	50.804	0.341	5.31	5.520	−5.774	16.392
MR	0.341	51.248	0.735	2.04	5.988	−9.980	14.060
LogP_o/w_	3.890	64.305	<0.01	1.18	0.304	0.575	1.789
nRB	−0.189	51.222	0.851	0	1	−1.386	1.147
nHA	−0.831	52.776	0.410	0	0	−1.114	0.462
nHD	0.520	54.216	0.605	0	0	−0.392	0.666
nRings	0.860	60.475	0.393	0	0	−0.254	0.638
nRigidB	1.420	54.802	0.161	2	1	−0.759	4.442
nAtoms	−1.094	53.416	0.279	−3	3	−7.969	2.344

Equal variances not assumed.

**Table 19:** *T*-test for equality of distributions (independent-samples Kolmogorov–Smirnov test).

Independent-Samples Kolmogorov–Smirnov Test				
Molecular Descriptors	Most Extreme Differences			Significance
	Absolute (D)	Positive	Negative	Two-Sided (*p*)
Molecular weight	0.201	0.181	−0.201	0.514
TPSA	0.228	0.228	−0.103	0.352
MR	0.147	0.147	−0.095	0.866
LogP_o/w_	0.406	0.406	0.000	0.008
nRB	0.165	0.078	−0.165	0.753
nHA	0.166	0.010	−0.166	0.748
nHD	0.071	0.071	−0.043	1.000
nRings	0.115	0.115	−0.026	0.981
nRigidB	0.210	0.210	−0.043	0.453
nAtoms	0.156	0.061	−0.156	0.815

Total N = 29 + 39= 68.

## Data Availability

The data are available by the authors and through literature.

## Supplementary Materials

The following supporting information can be downloaded at: https://www.mdpi.com/article/10.3390/ijms262010207/s1.
