# Supplementary material for: Using a Novel Consensus-Based Chemoinformatics Approach to Predict ADMET Properties and Druglikeness of Tyrosine Kinase Inhibitors
Source: Int J Mol Sci. 2025 Oct 20;26(20):10207. doi: 10.3390/ijms262010207 (PMC12563095; doi:10.3390/ijms262010207)
Supplement: Supplementary file 1 [file ijms-26-10207-s001.zip › ijms-3917525-supplementary.pdf]

Supplementary Materials

Table S1. Druglikeness evaluation according to Lipinski, CMC-like, Veber, Egan, Muegge, MMDR-like and QED rules of potent tyrosine kinase inhibitors.

ID	Physicochemical Properties												Druglikeness							Score[1]
	Molecular weight (g/mol)	Molecular Volume (A^3)	TPSA (A^2)	Molar Refractivity	Log $P_{o/w}$	Log S (moles/L)	Num. rotatable bonds	Num. H-bond acceptors	Num. H-bond donors	Num. Rings	Num. Rigid bonds	Num. atoms	Lipinski Rule	Ghose / CMC-like rule	Veber Rule	Egan Rule	Muegge Rule	MMDR-like Rules	QED (ADMETlab)	
**AIK.1**	435,46	430,63	94,33	126,81	2,81	-4,28	8	8	2	4	22	61	PASS	PASS	PASS	PASS	PASS	PASS	0,591	6,591
**TKI.19**	366,35	369,49	78,58	103,62	3,25	-4,42	8	6	2	3	19	49	PASS	PASS	PASS	PASS	PASS	PASS	0,496	6,496
**TKI.20a**	447,42	420,87	88,04	125,32	4,80	-5,84	9	7	1	4	24	53	PASS	PASS	PASS	PASS	PASS	PASS	0,317	6,317
**TKI.20b**	463,41	430,28	107,76	127,34	4,08	-5,32	9	8	2	4	24	54	PASS	PASS	PASS	PASS	PASS	PASS	0,3	6,3
**TKI.10**	297,57	281,63	44,65	82,83	4,47	-5,21	2	3	1	3	19	33	PASS	PASS	PASS	PASS	PASS	mid-structure	0,913	5,913
**DDK8**	230,27	231,76	51,97	70,08	3,06	-3,22	2	3	2	3	16	35	PASS	PASS	PASS	PASS	PASS	mid-structure	0,833	5,833
**TKI.1**	329,28	302,63	74,11	89,01	3,17	-4,17	4	6	2	3	17	40	PASS	PASS	PASS	PASS	PASS	mid-structure	0,762	5,762
**TKI.21b**	379,31	361,06	114,14	113,33	2,82	-4,95	4	7	1	4	26	42	PASS	PASS	PASS	PASS	PASS	mid-structure	0,743	5,743
**TKI.5**	405,35	364,83	111,11	116,69	3,26	-4,60	4	8	2	5	28	44	PASS	PASS	PASS	PASS	PASS	mid-structure	0,646	5,646
**TKI.16**	325,32	307,90	94,14	90,51	2,21	-3,34	4	7	3	4	22	43	PASS	PASS	PASS	PASS	PASS	mid-structure	0,642	5,642
**TKI.14b**	342,31	289,05	92,90	89,90	2,65	-3,93	4	6	1	4	21	33	PASS	PASS	PASS	PASS	PASS	mid-structure	0,574	5,574
**TKI.9**	402,39	400,49	96,33	117,82	2,21	-3,80	5	7	2	4	25	52	PASS	PASS	PASS	PASS	PASS	mid-structure	0,531	5,531
**TKI.14a**	360,36	318,85	56,84	102,17	4,94	-5,44	3	5	0	5	26	37	PASS	PASS	PASS	PASS	PASS	mid-structure	0,446	5,446

**TKI.2b**	387,37	379,51	88,96	114,02	4,90	-5,61	5	6	3	4	23	50	PASS	PASS	PASS	PASS	PASS	mid-structure	0,442	5,442
**TKI.4**	492,53	495,32	93,92	145,45	4,11	-3,45	7	8	0	5	32	65	PASS	FAIL	PASS	PASS	PASS	PASS	0,385	5,385
**TKI.2a**	386,39	380,26	91,65	117,05	4,96	-5,50	5	5	4	4	23	51	PASS	PASS	PASS	PASS	PASS	mid-structure	0,384	5,384
**TKI.8**	505,05	447,17	90,63	137,78	4,91	-5,93	7	7	2	5	30	53	1 violation	FAIL	PASS	PASS	PASS	PASS	0,328	4,828
**TKI.21a**	396,39	373,80	91,66	117,08	5,38	-6,18	6	6	2	4	25	45	1 violation	PASS	PASS	PASS	FAIL	PASS	0,323	4,823
**TKI.17**	524,46	473,18	130,69	143,30	3,18	-4,15	8	10	2	6	33	58	1 violation	FAIL	PASS	PASS	PASS	PASS	0,301	4,801
**TKI.18**	573,83	515,40	128,35	161,20	4,70	-6,11	6	9	1	5	34	64	1 violation	FAIL	PASS	PASS	PASS	PASS	0,292	4,792
**TKI.13a**	579,83	548,17	121,77	171,25	4,45	-5,59	6	8	3	6	38	68	1 violation	FAIL	PASS	PASS	PASS	PASS	0,269	4,769
**TKI.13b**	595,83	557,73	130,76	172,78	4,01	-5,52	7	9	3	6	38	69	1 violation	FAIL	PASS	PASS	PASS	PASS	0,254	4,754
**TKI.6**	393,43	359,82	78,09	112,80	5,49	-5,69	2	6	1	6	30	46	1 violation	PASS	PASS	PASS	FAIL	mid-structure	0,507	4,007
**TKI.11**	598,16	524,59	103,15	159,17	4,92	-5,43	12	8	2	5	30	68	1 violation	FAIL	FAIL	PASS	PASS	PASS	0,164	3,664
**TKI.7a**	560,45	513,75	121,41	148,62	5,34	-5,82	10	8	4	4	28	64	FAIL	FAIL	PASS	PASS	FAIL	PASS	0,191	3,191
**AIK.3**	433,32	385,87	65,75	112,49	5,69	-5,77	3	6	1	5	27	46	1 violation	FAIL	PASS	PASS	FAIL	mid-structure	0,381	2,881
**TKI.15**	437,46	434,40	76,96	133,94	6,26	-6,36	6	5	3	5	30	60	1 violation	FAIL	PASS	FAIL	FAIL	PASS	0,333	2,833
**TKI.3**	461,80	408,61	85,43	130,57	6,18	-6,54	6	6	2	5	29	52	1 violation	FAIL	PASS	FAIL	FAIL	PASS	0,294	2,794
**TKI.7b**	617,49	566,64	149,85	161,23	4,78	-5,41	13	10	5	4	29	71	1 violation	FAIL	FAIL	FAIL	FAIL	PASS	0,152	1,652

[1] Scoring index: Pass (green)=1, Fail (red)=0, 1 violation (yellow)=0.5, mid-structure (blue)=0, Minimum acceptable score=3.501.

QED (Quantitative Estimate of Druglikeness) parameter is used to quantify compound quality, applying the concept of desirability to provide a quantitative metric for assessing druglikeness. The QED values can range between zero (all properties unfavorable) and one (all properties favorable). For QED calculations, eight widely used molecular properties were selected based on published precedence for their relevance in determining druglikeness: molecular weight (MW), octanol-water partition coefficient (ALOGP), number of hydrogen bond donors (HBD), number of hydrogen bond acceptors (HBA), molecular polar surface area (PSA), number of rotatable bonds (ROTB), number of aromatic rings (AROM), and number of structural alerts (ALERTS) [7].

Table S2. Criteria for the accomplishment of Medicinal's Chemistry (Leadlikeness, GSK, PAINS, Brenk) rules in potent tyrosine kinase inhibitors.

ID	Physicochemical Properties												Medicinal Chemistry				Score[1]
	Molecular weight (g/mol)	Molecular Volume (A³)	TPSA (A²)	Molar Refractivity	Log $P_{o/w}$	Log S (moles/L)	Num. rotatable bonds	Num. H-bond acceptors	Num. H-bond donors	Num. Rings	Num. Rigid bonds	Num. atoms	Leadlikeness	GSK Rule	PAINS (SwissADME)	Brenk (SwissADME)	
**TKI.1**	329,28	302,63	74,11	89,01	3,17	-4,17	4	6	2	3	17	40	PASS	PASS	PASS	PASS	4
**TKI.14b**	342,31	289,05	92,90	89,90	2,65	-3,93	4	6	1	4	21	33	PASS	PASS	PASS	PASS	4
**TKI.16**	325,32	307,90	94,14	90,51	2,21	-3,34	4	7	3	4	22	43	PASS	PASS	PASS	PASS	4
**TKI.21b**	379,31	361,06	114,14	113,33	2,82	-4,95	4	7	1	4	26	42	FAIL	PASS	PASS	PASS	3
**DDK8**	230,27	231,76	51,97	70,08	3,06	-3,22	2	3	2	3	16	35	FAIL	PASS	PASS	PASS	3
**TKI.19**	366,35	369,49	78,58	103,62	3,25	-4,42	8	6	2	3	19	49	FAIL	PASS	PASS	FAIL	2
**AIK.1**	435,46	430,63	94,33	126,81	2,81	-4,28	8	8	2	4	22	61	FAIL	FAIL	PASS	PASS	2
**TKI.2a**	386,39	380,26	91,65	117,05	4,96	-5,50	5	5	4	4	23	51	FAIL	FAIL	PASS	PASS	2
**TKI.2b**	387,37	379,51	88,96	114,02	4,90	-5,61	5	6	3	4	23	50	FAIL	FAIL	PASS	PASS	2
**TKI.4**	492,53	495,32	93,92	145,45	4,11	-3,45	7	8	0	5	32	65	FAIL	FAIL	PASS	PASS	2
**TKI.14a**	360,36	318,85	56,84	102,17	4,94	-5,44	3	5	0	5	26	37	FAIL	FAIL	PASS	PASS	2
**TKI.8**	505,05	447,17	90,63	137,78	4,91	-5,93	7	7	2	5	30	53	FAIL	FAIL	PASS	PASS	2
**TKI.6**	393,43	359,82	78,09	112,80	5,49	-5,69	2	6	1	6	30	46	FAIL	FAIL	PASS	PASS	2
**TKI.17**	524,46	473,18	130,69	143,30	3,18	-4,15	8	10	2	6	33	58	FAIL	FAIL	PASS	PASS	2
**TKI.15**	437,46	434,40	76,96	133,94	6,26	-6,36	6	5	3	5	30	60	FAIL	FAIL	PASS	PASS	2
**TKI.11**	598,16	524,59	103,15	159,17	4,92	-5,43	12	8	2	5	30	68	FAIL	FAIL	PASS	PASS	2
**TKI.20a**	447,42	420,87	88,04	125,32	4,80	-5,84	9	7	1	4	24	53	FAIL	FAIL	PASS	FAIL	1
**TKI.9**	402,39	400,49	96,33	117,82	2,21	-3,80	5	7	2	4	25	52	FAIL	FAIL	PASS	FAIL	1

**TKI.10**	297,57	281,63	44,65	82,83	4,47	-5,21	2	3	1	3	19	33	FAIL	FAIL	FAIL	PASS	1
**TKI.13a**	579,83	548,17	121,77	171,25	4,45	-5,59	6	8	3	6	38	68	FAIL	FAIL	PASS	FAIL	1
**TKI.13b**	595,83	557,73	130,76	172,78	4,01	-5,52	7	9	3	6	38	69	FAIL	FAIL	PASS	FAIL	1
**TKI.21a**	396,39	373,80	91,66	117,08	5,38	-6,18	6	6	2	4	25	45	FAIL	FAIL	PASS	FAIL	1
**AIK.3**	433,32	385,87	65,75	112,49	5,69	-5,77	3	6	1	5	27	46	FAIL	FAIL	PASS	FAIL	1
**TKI.7a**	560,45	513,75	121,41	148,62	5,34	-5,82	10	8	4	4	28	64	FAIL	FAIL	PASS	FAIL	1
**TKI.3**	461,80	408,61	85,43	130,57	6,18	-6,54	6	6	2	5	29	52	FAIL	FAIL	PASS	FAIL	1
**TKI.7b**	617,49	566,64	149,85	161,23	4,78	-5,41	13	10	5	4	29	71	FAIL	FAIL	PASS	FAIL	1
**TKI.20b**	463,41	430,28	107,76	127,34	4,08	-5,32	9	8	2	4	24	54	FAIL	FAIL	FAIL	FAIL	0
**TKI.5**	405,35	364,83	111,11	116,69	3,26	-4,60	4	8	2	5	28	44	FAIL	FAIL	FAIL	FAIL	0
**TKI.18**	573,83	515,40	128,35	161,20	4,70	-6,11	6	9	1	5	34	64	FAIL	FAIL	FAIL	FAIL	0

[1] Scoring index: Pass (green)=1, Fail (red)=0, Minimum acceptable score=2.

PAINS is a filter used for the removal of Pan Assay Interference Compounds (PAINS), that is, compounds being frequent hitters (also known as promiscuous compounds), which generate false-positive signals in biochemical assays, in contrast to compounds that are genuinely active against the target [27]. Brenk's structural rules include the absence of unwanted functionalities (e.g., chemical moieties known to be toxic, unstable, dye), lead-like properties, and limited complexity [28].

Table S3. Bioavailability evaluation (Caco-2 Permeability, HIA, MDCK premeability, Pgp-substrate/inhibitor) of potent tyrosine kinase inhibitors.

ID	Bioavailability					Score[1]
	Caco-2 Permeability	Human Intestinal Absorption	MDCK Permeability	Pgp-substrate	Pgp-inhibitor	
TKI.16	high	high	high	No	No	5
TKI.14a	high	high	high	No	No	5
TKI.14b	high	high	high	No	No	5
DDK.8	high	high	high	No	No	5
TKI.10	high	high	high	No	Yes	4
TKI.19	high	high	high	No	Yes	4
TKI.5	high	high	high	No	Yes	4
TKI.21a	high	high	high	No	Yes	4
TKI.21b	high	high	high	No	Yes	4
TKI.6	high	high	high	No	Yes	4
TKI.1	high	high	high	No	Yes	4
TKI.9	high	high	high	No	Yes	4
AIK.3	high	high	high	No	Yes	4
TKI.20a	high	high	moderate	No	Yes	3,5
TKI.3	high	high	moderate	No	Yes	3,5
TKI.4	moderate	high	high	Yes	Yes	3,5
AIK.1	high	high	moderate	Yes	Yes	3,5
TKI.20b	moderate	high	moderate	No	Yes	3
TKI.2a	moderate	high	moderate	No	Yes	3
TKI.2b	high	high	low	No	Yes	3
TKI.7a	moderate	high	moderate	Yes	Yes	3
TKI.8	moderate	high	low	No	Yes	2,5
TKI.13b	moderate	high	low	Yes	Yes	2,5
TKI.18	moderate	high	low	No	Yes	2,5
TKI.11	moderate	high	low	Yes	Yes	2,5
TKI.13a	moderate	high	low	Yes	Yes	2,5
TKI.15	moderate	high	low	No	Yes	2,5
TKI.17	moderate	high	low	No	Yes	2,5
TKI.7b	moderate	high	low	Yes	Yes	2,5

[1] Scoring index: high (green)=1, moderate (yellow)=0.5, low (red)=0, No/No (green/red)=2, No/Yes (green/green)=1,Yes/Yes (red/green)=1, Yes/No (red/red)=0, Minimum acceptable score=2.51.

Table S4. Distribution (PPB) and Excretion (Total Clearance) evaluation of potent tyrosine kinase inhibitors.

ID	Distribution	ID	Excretion
	Plasma Protein Binding (PPB)[1]		Total Clearance
TKI.16	low	TKI.1	low
AIK.1	low	TKI.2a	low
TKI.6	high	TKI.2b	low
TKI.9	high	TKI.3	low
TKI.19	high	TKI.4	low
DDK.8	high	TKI.5	low

**TKI.1**	**high**	**TKI.6**	**low**
**TKI.2a**	**high**	**TKI.7a**	**low**
**TKI.2b**	**high**	**TKI.7b**	**low**
**TKI.3**	**high**	**TKI.8**	**low**
**TKI.4**	**high**	**TKI.9**	**low**
**TKI.5**	**high**	**TKI.10**	**low**
**TKI.7a**	**high**	**TKI.11**	**low**
**TKI.7b**	**high**	**TKI.13a**	**low**
**TKI.8**	**high**	**TKI.13b**	**low**
**TKI.10**	**high**	**TKI.14a**	**low**
**TKI.11**	**high**	**TKI.14b**	**low**
**TKI.13a**	**high**	**TKI.15**	**low**
**TKI.13b**	**high**	**TKI.16**	**low**
**TKI.14a**	**high**	**TKI.17**	**low**
**TKI.14b**	**high**	**TKI.18**	**low**
**TKI.15**	**high**	**TKI.19**	**high**
**TKI.17**	**high**	**TKI.20a**	**low**
**TKI.18**	**high**	**TKI.20b**	**low**
**TKI.20a**	**high**	**TKI.21a**	**low**
**TKI.20b**	**high**	**TKI.21b**	**low**
**TKI.21a**	**high**	**AIK.1**	**low**
**TKI.21b**	**high**	**AIK.3**	**low**
**AIK.3**	**high**	**DDK.8**	**high**

[1] Colour index: high =red, low =green, Acceptable colour =green.

Table S5. Toxicity evaluation (Carcinogenic potential & Organ toxicity) of potent tyrosine kinase inhibitors.

**NAME**	**TOXICITY**				**Score[1]**
	**Carcinogenic potential**		**Organ toxicity**		
	**Ames test**	**Carcinogencity (rat)**	**hERG Blockers**	**Hepatotoxicity**	
**TKI.18**	negative	negative	inactive	negative	4
**TKI.13b**	negative	negative	inactive	negative	4
**TKI.19**	positive	negative	inactive	negative	3
**DDK8**	negative	negative	inactive	positive	3
**TKI.8**	negative	negative	inactive	positive	3
**TKI.4**	negative	negative	active	negative	3
**TKI.21b**	negative	negative	inactive	positive	3
**TKI.20b**	negative	negative	inactive	positive	3
**TKI.13a**	negative	negative	active	negative	3
**TKI.17**	negative	negative	active	positive	2
**TKI.10**	positive	positive	inactive	negative	2

TKI.6	positive	negative	inactive	positive	2
TKI.7b	negative	negative	active	positive	2
TKI.9	positive	negative	active	negative	2
TKI.11	negative	negative	active	positive	2
TKI.14a	positive	negative	inactive	positive	2
TKI.7a	negative	negative	active	positive	2
TKI.20a	positive	negative	inactive	positive	2
TKI.3	positive	negative	inactive	positive	2
AIK.3	positive	negative	active	negative	2
TKI.16	positive	positive	inactive	positive	1
TKI.5	positive	positive	inactive	positive	1
TKI.14b	positive	positive	inactive	positive	1
TKI.1	positive	positive	inactive	positive	1
TKI.2a	positive	negative	active	positive	1
TKI.21a	positive	positive	inactive	positive	1
TKI.15	positive	negative	active	positive	1
TKI.2b	positive	negative	active	positive	1
AIK.1	positive	positive	active	positive	0

[1] Scoring index: Positive (red)=0, Negative (green)=1, Active (red)=0, Inactive (green)=1, Minimum acceptable score=2.01.

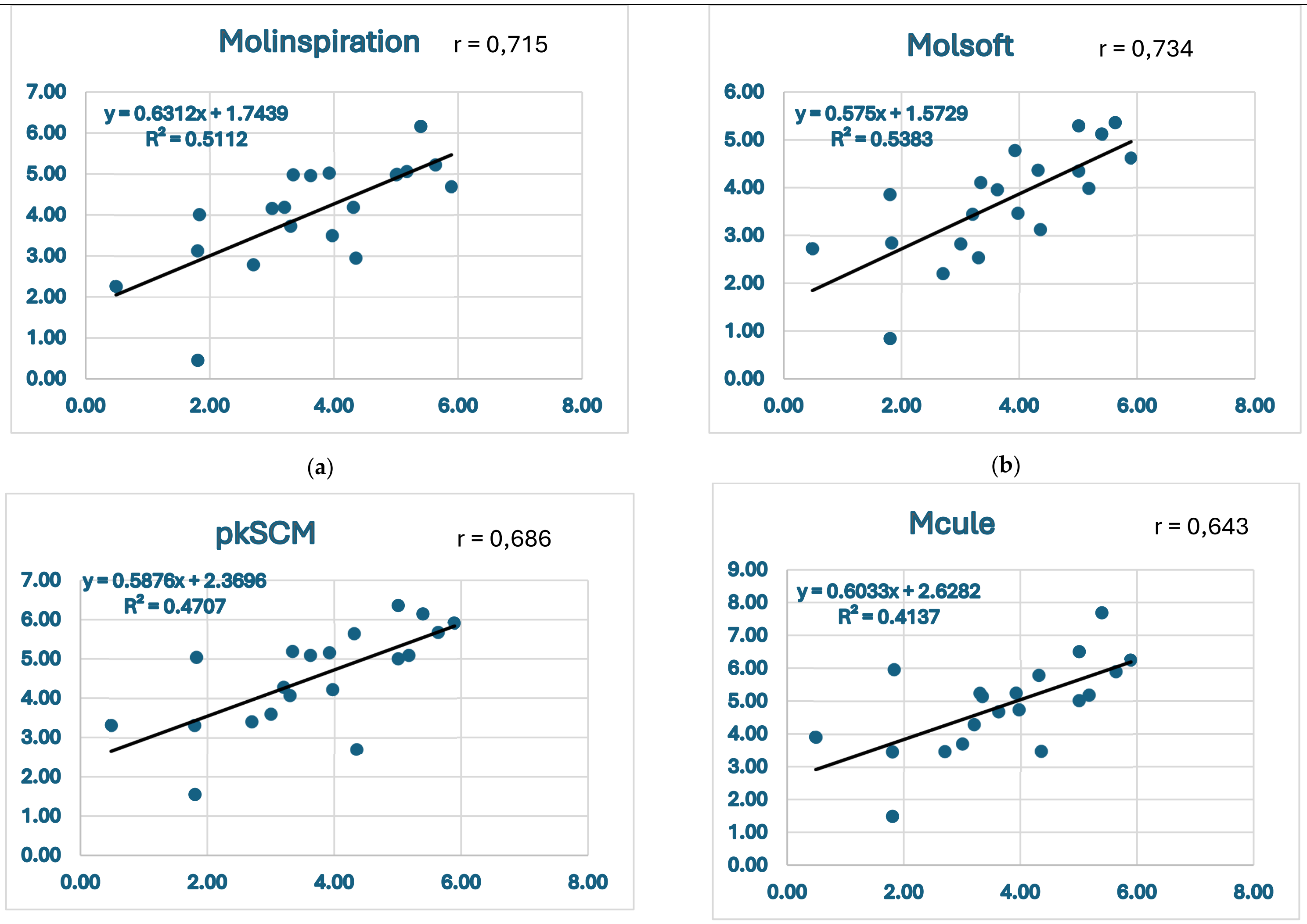

(a) (b) (c) (d)

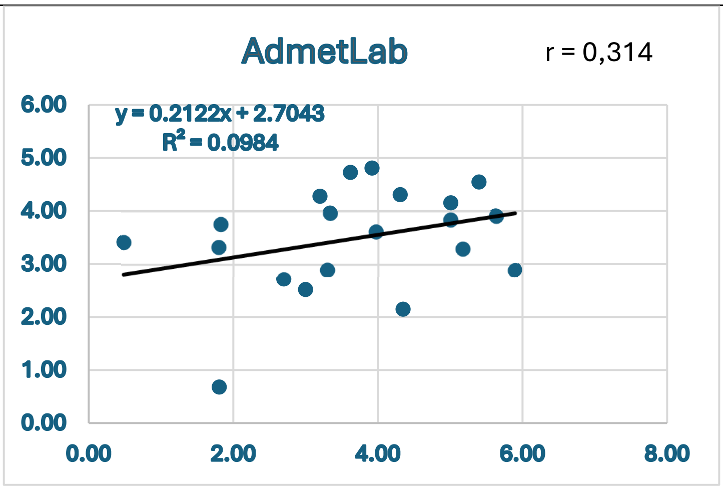

(e)

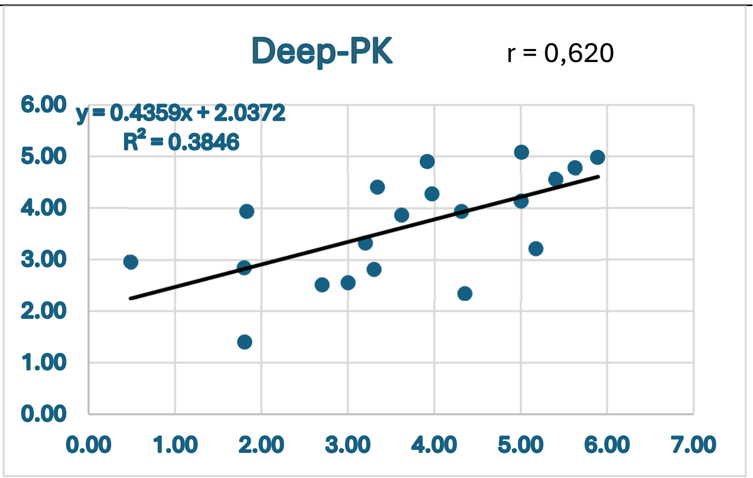

(f)

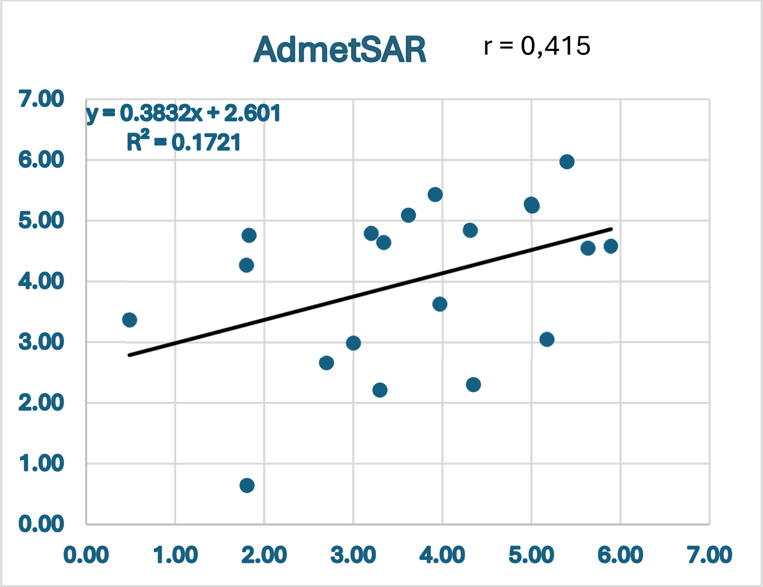

(g)

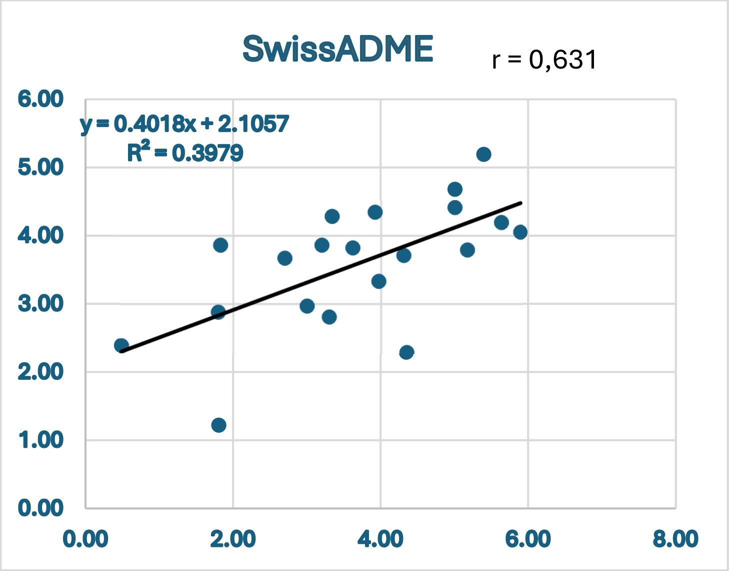

(h)

Figure S1. Regression analysis for LogP value of: (a) Molinspiration; (b) Molsoft; (c) pKSCM; (d) Mcule; (e) AdmetLab; (f) Deep-PK; (g) Hepatotxicity; (h) SwissADME.

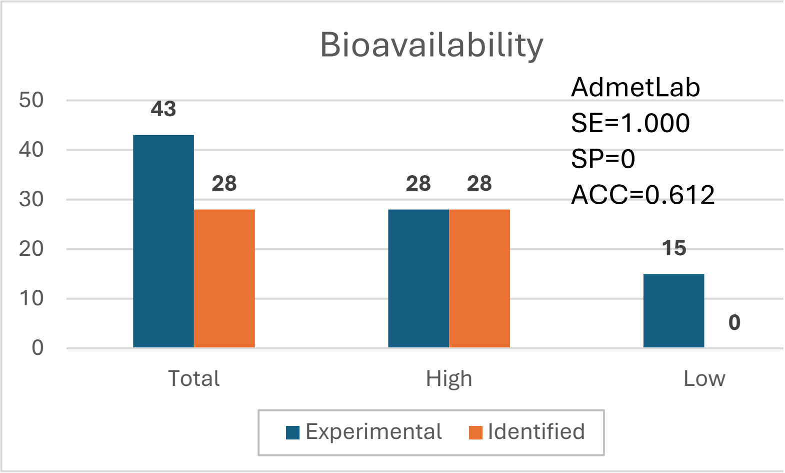

(a)

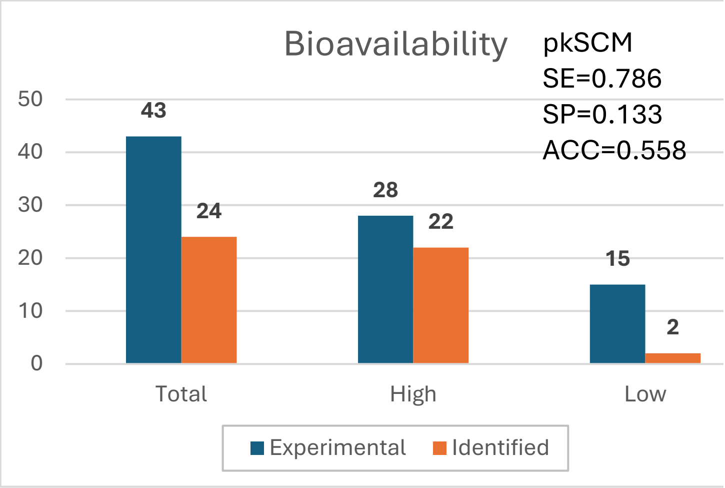

(b)

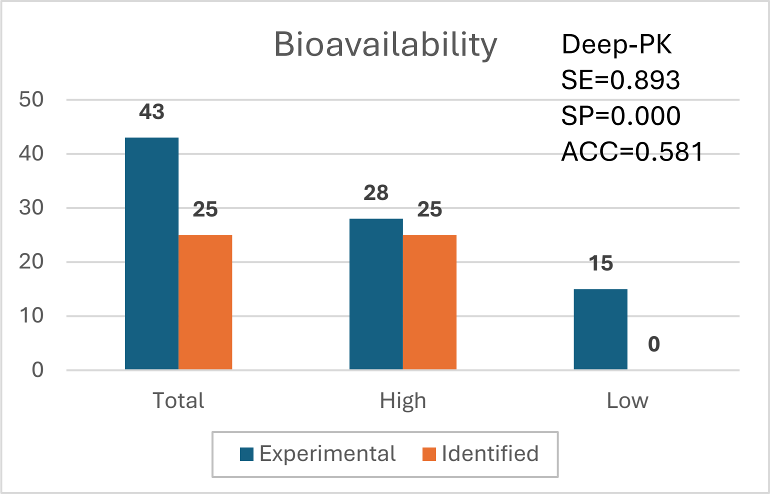
Bioavailability
Deep-PK
SE=0.893
SP=0.000
ACC=0.581
50
40
30
20
10
0
43
25
28
25
15
0
Total
High
Low
Experimental
Identified

(c)

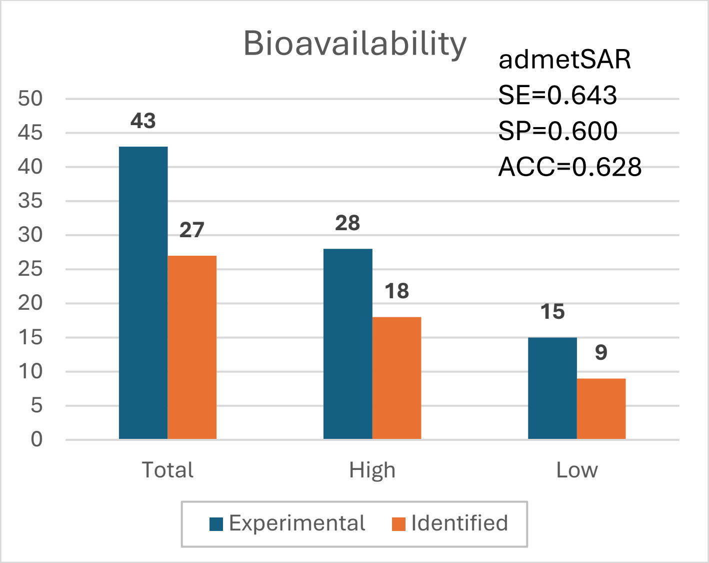
Bioavailability
admetSAR
SE=0.643
SP=0.600
ACC=0.628
50
45
40
35
30
25
20
15
10
5
0
43
27
28
18
15
9
Total
High
Low
Experimental
Identified

(d)

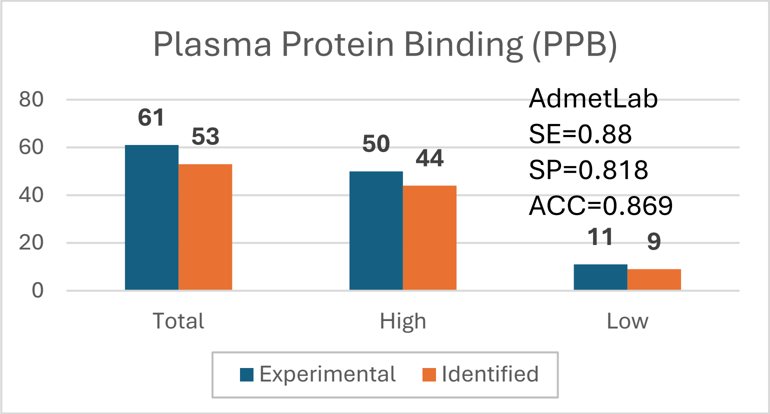
Plasma Protein Binding (PPB)
AdmetLab
SE=0.88
SP=0.818
ACC=0.869
80
60
40
20
0
61
53
50
44
11
9
Total
High
Low
Experimental
Identified

(e)

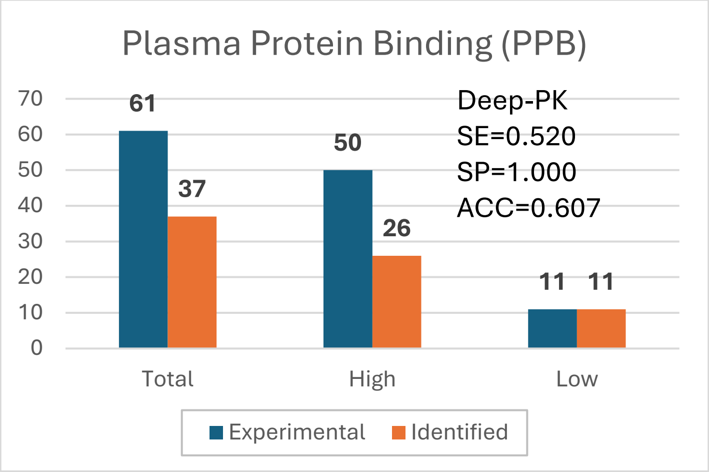
Plasma Protein Binding (PPB)
Deep-PK
SE=0.520
SP=1.000
ACC=0.607
70
60
50
40
30
20
10
0
61
37
50
26
11
11
Total
High
Low
Experimental
Identified

(f)

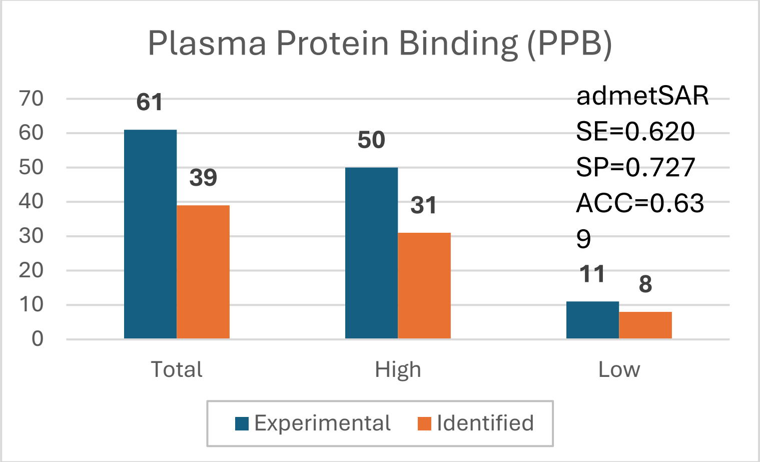
Plasma Protein Binding (PPB)
admetSAR
SE=0.620
SP=0.727
ACC=0.63
9
70
60
50
40
30
20
10
0
61
39
50
31
11
8
Total
High
Low
Experimental
Identified

(g)

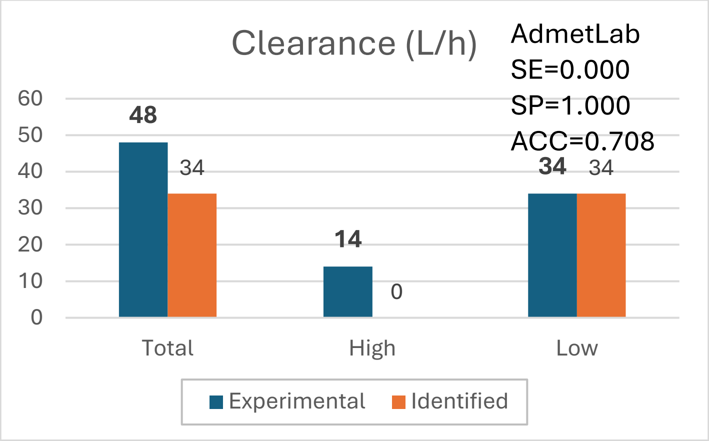
Clearance (L/h)
AdmetLab
SE=0.000
SP=1.000
ACC=0.708
60
50
40
30
20
10
0
48
34
14
0
34
34
Total
High
Low
Experimental
Identified

(h)

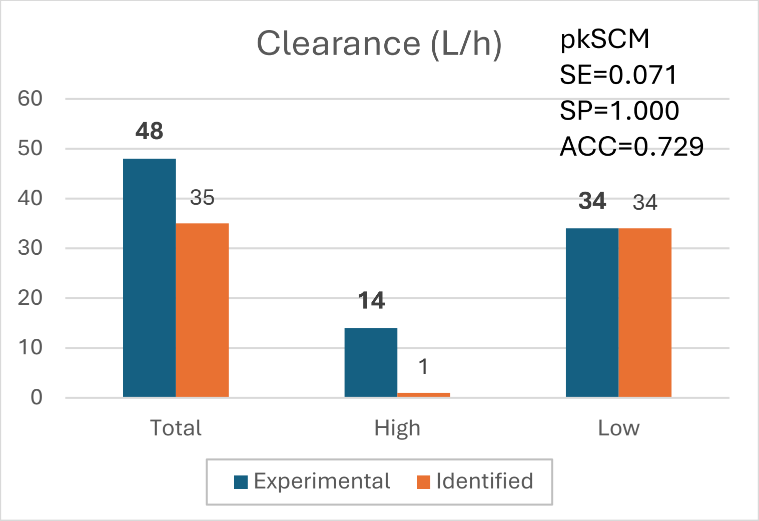
Clearance (L/h)
pkSCM
SE=0.071
SP=1.000
ACC=0.729
60
50
40
30
20
10
0
48
35
14
1
34
34
Total
High
Low
Experimental
Identified

(i)

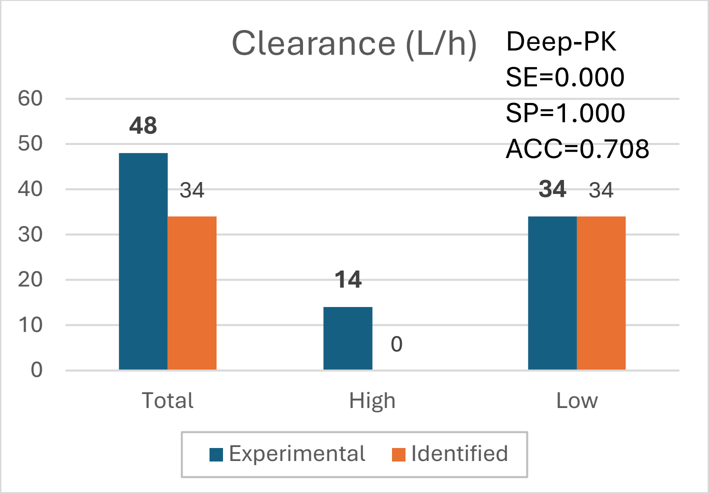
Clearance (L/h)
Deep-PK
SE=0.000
SP=1.000
ACC=0.708
60
50
40
30
20
10
0
48
34
14
0
34
34
Total
High
Low
Experimental
Identified

(j)

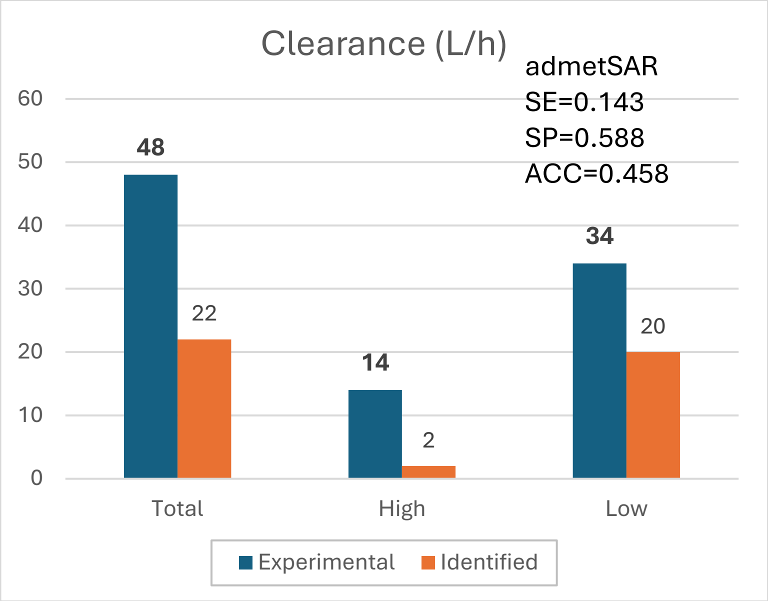
Clearance (L/h)
admetSAR
SE=0.143
SP=0.588
ACC=0.458
60
50
40
30
20
10
0
48
22
14
2
34
20
Total
High
Low
Experimental
Identified

(k)

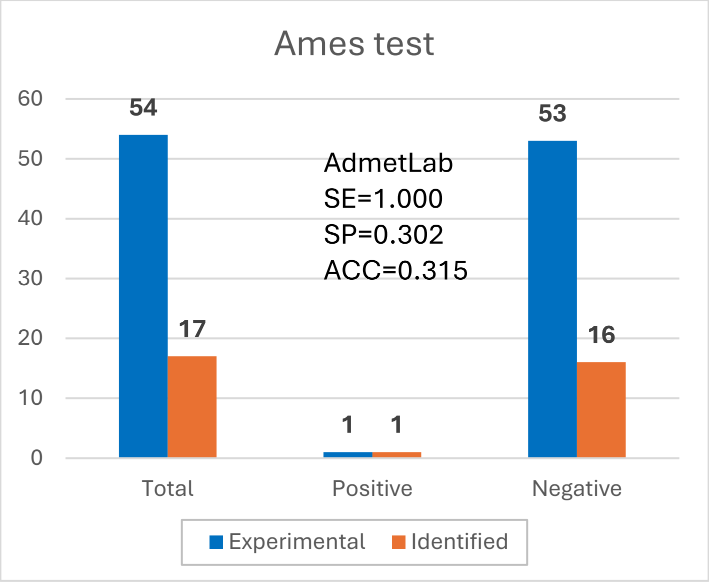
Ames test
AdmetLab
SE=1.000
SP=0.302
ACC=0.315
60
50
40
30
20
10
0
54
17
1
1
53
16
Total
Positive
Negative
Experimental
Identified

(l)

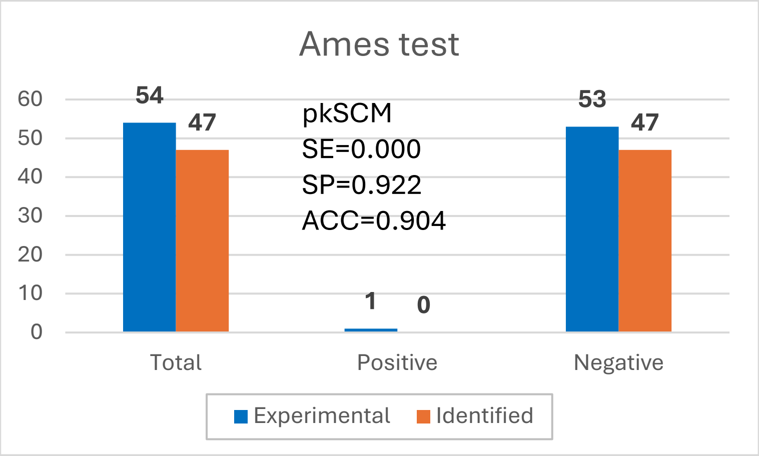
Ames test
pkSCM
SE=0.000
SP=0.922
ACC=0.904
60
50
40
30
20
10
0
54
47
1
0
53
47
Total
Positive
Negative
Experimental
Identified

(m)

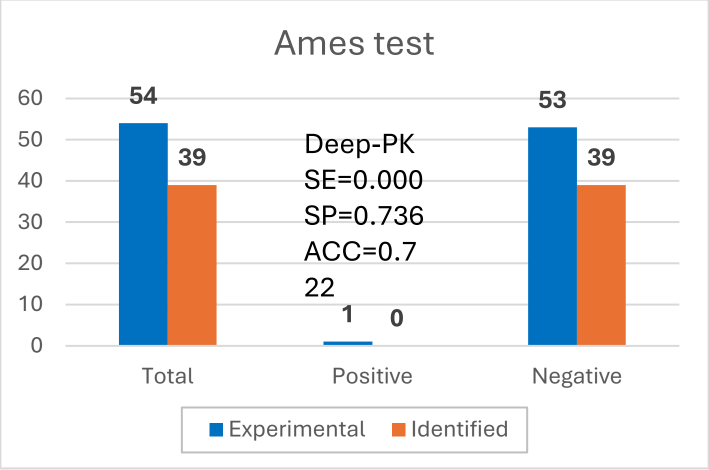
Ames test
Deep-PK
SE=0.000
SP=0.736
ACC=0.7
22
60
50
40
30
20
10
0
54
39
1
0
53
39
Total
Positive
Negative
Experimental
Identified

(n)

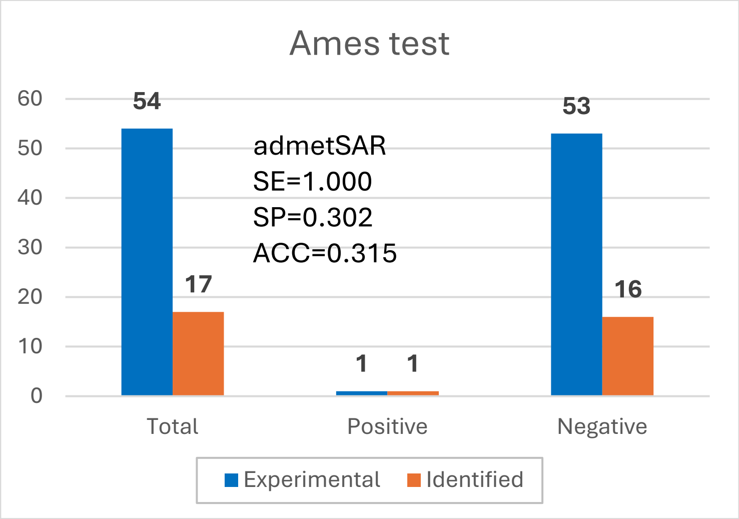
Ames test
60
50
40
30
20
10
0
54
17
1
1
53
16
admetSAR
SE=1.000
SP=0.302
ACC=0.315
Total
Positive
Negative
Experimental
Identified

(o)

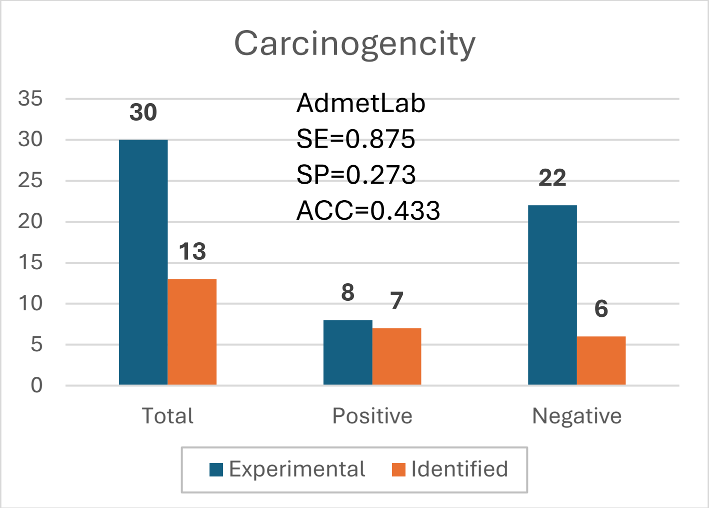
Carcinogencity
35
30
25
20
15
10
5
0
30
13
8
7
22
6
AdmetLab
SE=0.875
SP=0.273
ACC=0.433
Total
Positive
Negative
Experimental
Identified

(p)

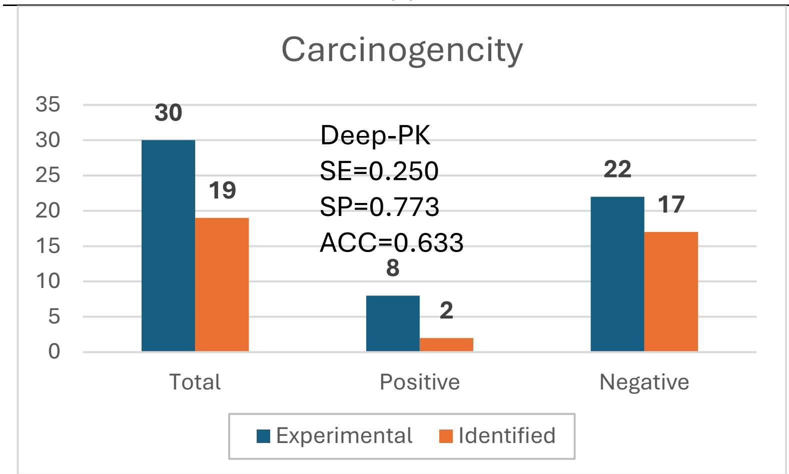
Carcinogencity
35
30
25
20
15
10
5
0
30
19
8
2
22
17
Deep-PK
SE=0.250
SP=0.773
ACC=0.633
Total
Positive
Negative
Experimental
Identified

(q)

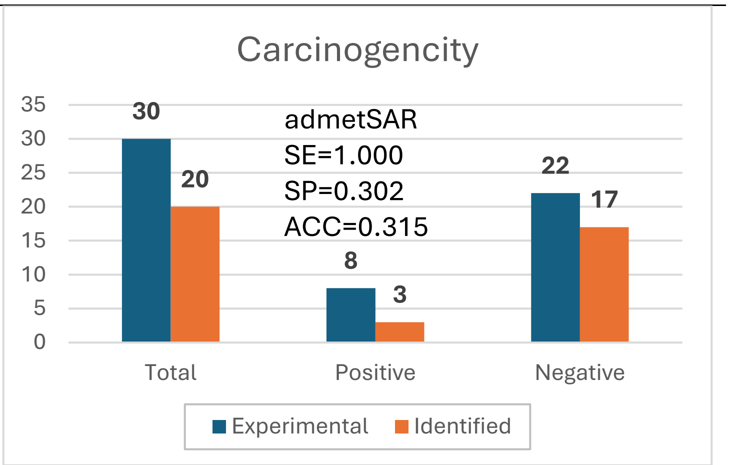
Carcinogencity
35
30
25
20
15
10
5
0
30
20
8
3
22
17
admetSAR
SE=1.000
SP=0.302
ACC=0.315
Total
Positive
Negative
Experimental
Identified

(r)

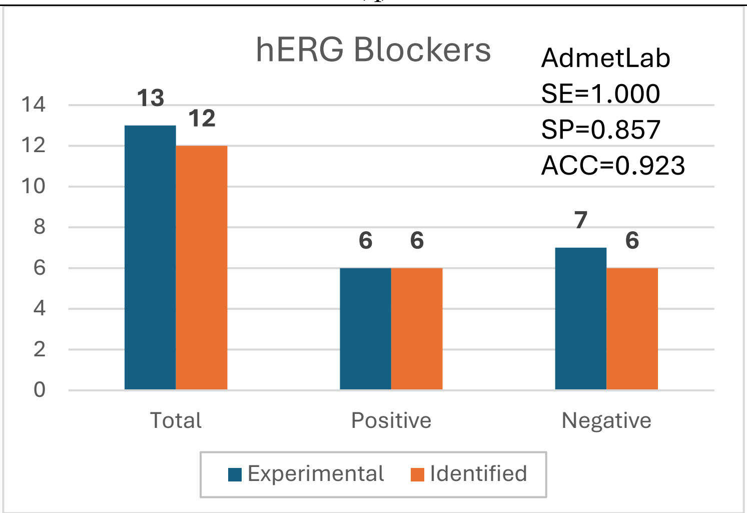
hERG Blockers
AdmetLab
SE=1.000
SP=0.857
ACC=0.923
14
12
10
8
6
4
2
0
13
12
6
6
7
6
Total
Positive
Negative
Experimental
Identified

(s)

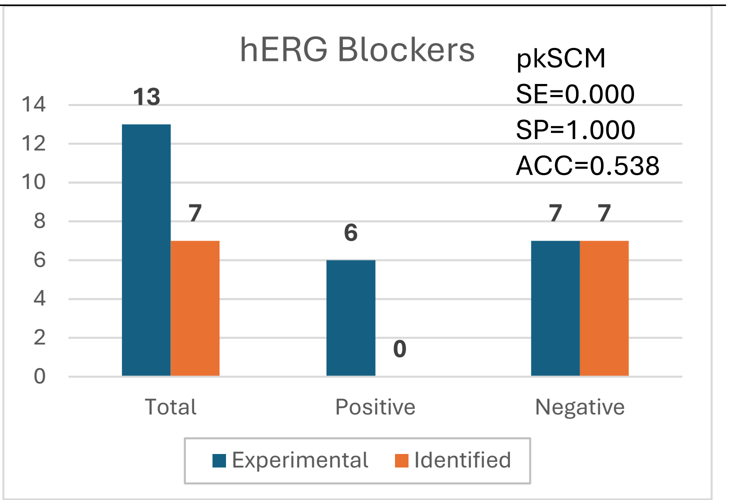
hERG Blockers
pkSCM
SE=0.000
SP=1.000
ACC=0.538
14
12
10
8
6
4
2
0
13
7
6
0
7
7
Total
Positive
Negative
Experimental
Identified

(t)

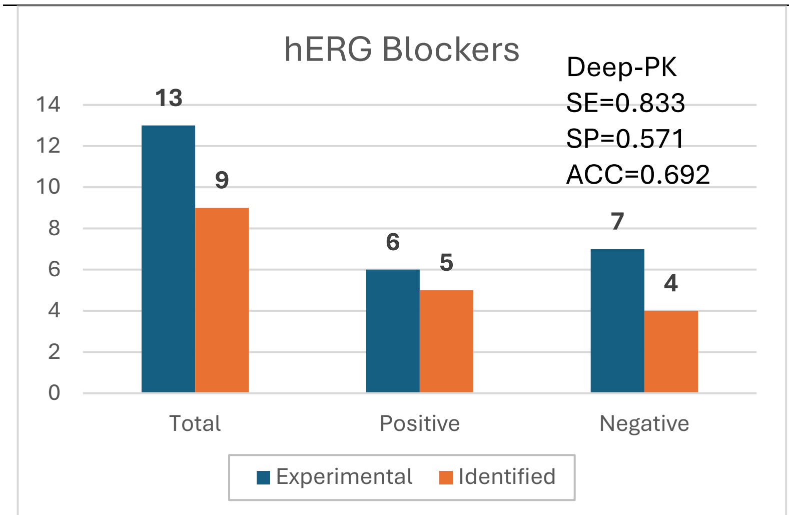

(u)

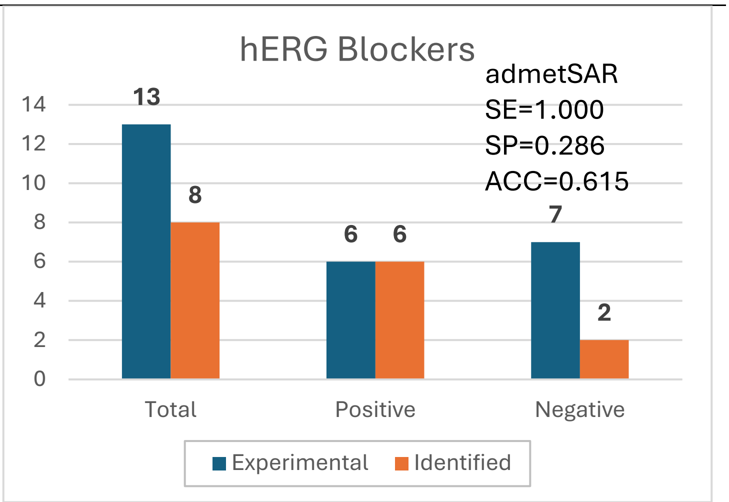

(v)

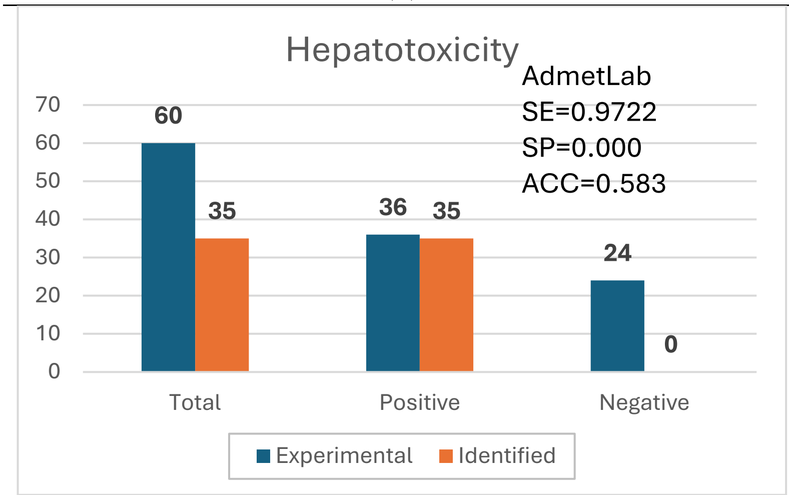

(w)

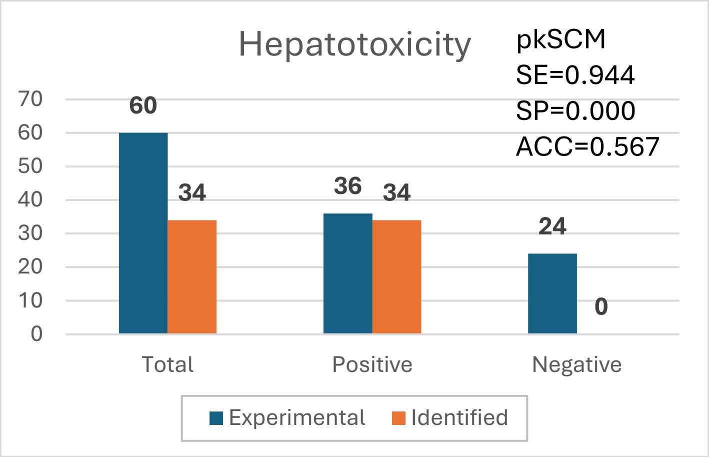

(x)

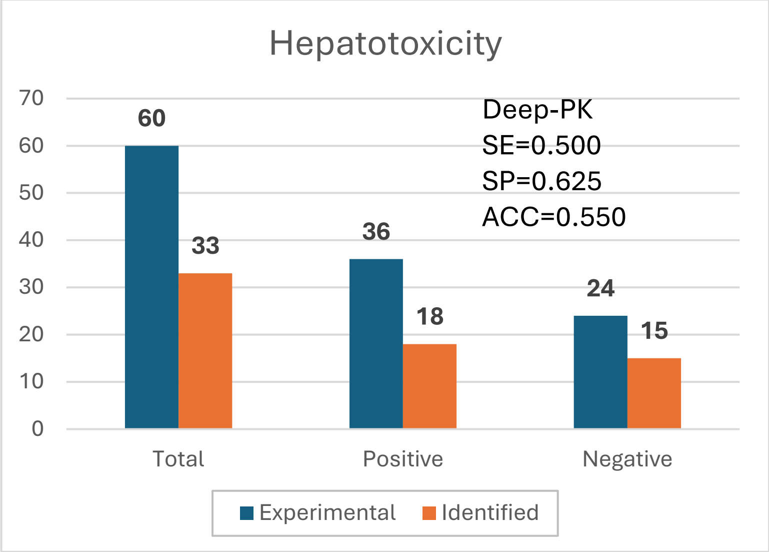

(y)

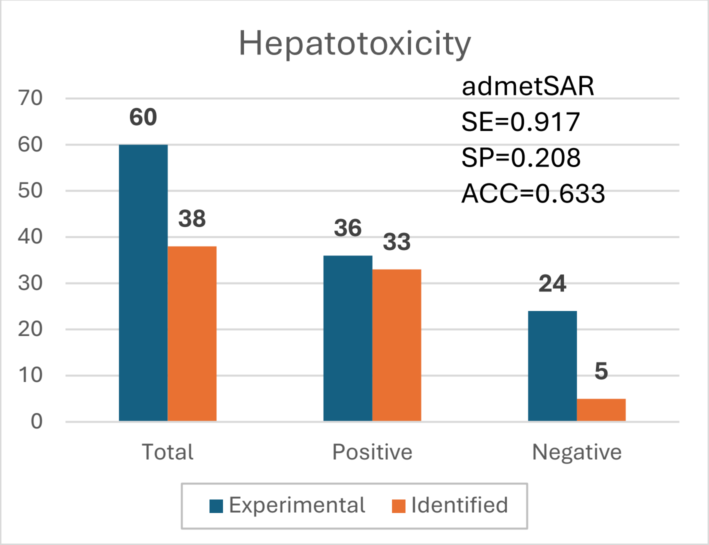

(z)

Figure S2. Bar graphical representations and evaluation of (**a-d**) Bioavailability; (**e-g**) Plasma Protein Binding; (**h-k**) Clearance; (**l-o**) Ames test; (**p-r**) Carcinogenicity; (**s-v**) hERG; (**w-z**) Hepatotxicity assessments, showing Experimental Total=N, Identified high/positive =TP, Identified low/negative =TN, Experimental low/negative – Identified low/negative =FP, Experimental high/positive – Identified high/positive =FN.

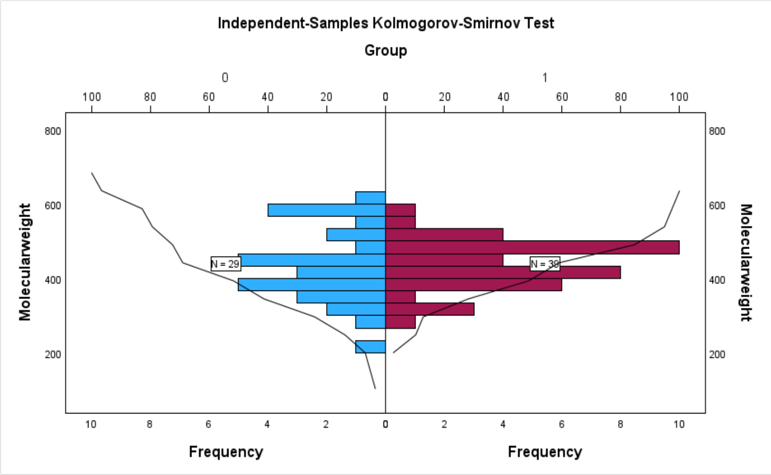
Independent-Samples Kolmogorov-Smirnov Test
Group
0
1
Molecularweight
N = 29
N = 39
Frequency
Frequency

(a)

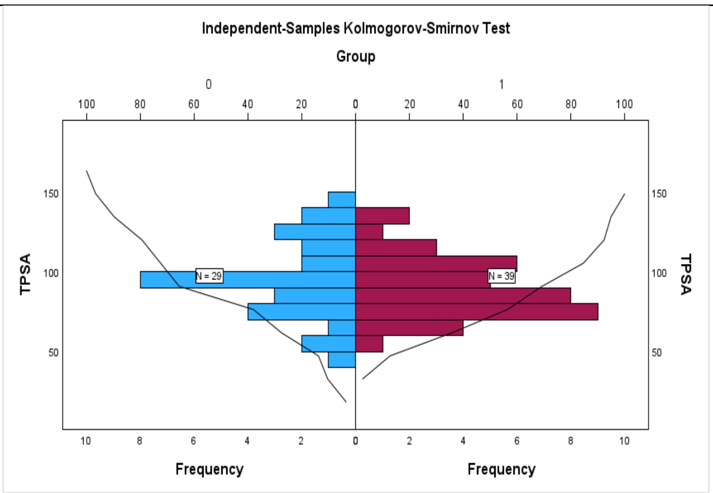
Independent-Samples Kolmogorov-Smirnov Test
Group
0
1
TPSA
N = 29
N = 39
Frequency
Frequency

(b)

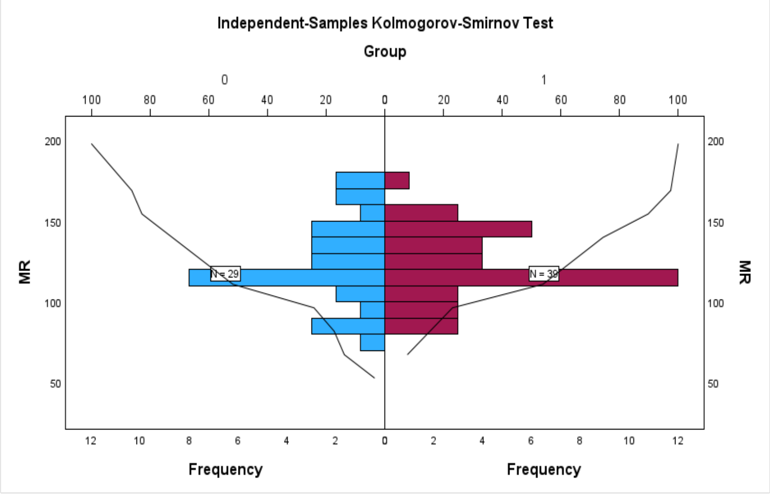
Independent-Samples Kolmogorov-Smirnov Test
Group
0
1
MR
N = 29
N = 39
Frequency
Frequency

(c)

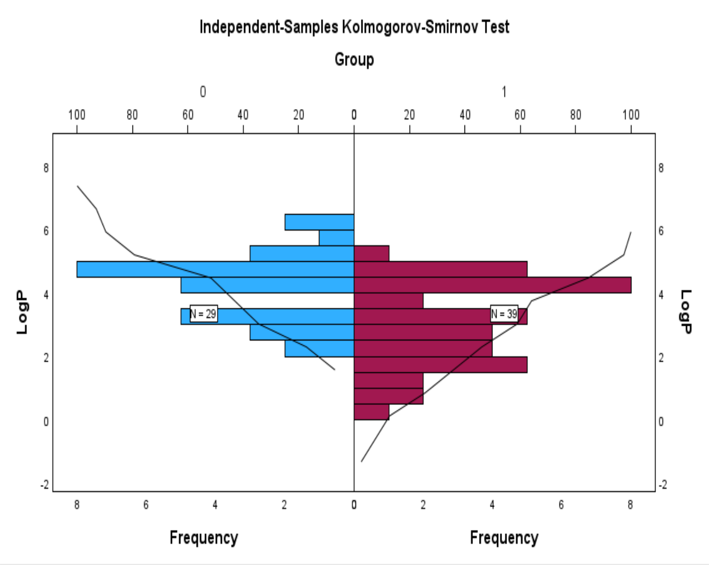
Independent-Samples Kolmogorov-Smirnov Test
Group
0
1
LogP
N = 29
N = 39
Frequency
Frequency

(d)

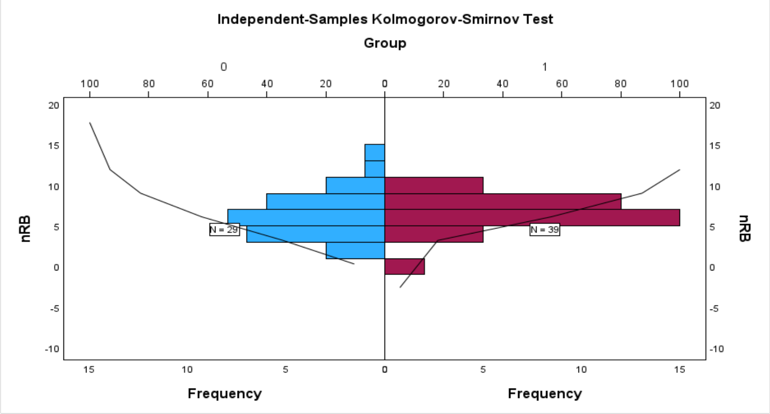
Independent-Samples Kolmogorov-Smirnov Test
Group
0
1
nRB
N = 29
N = 39
Frequency
Frequency

(e)

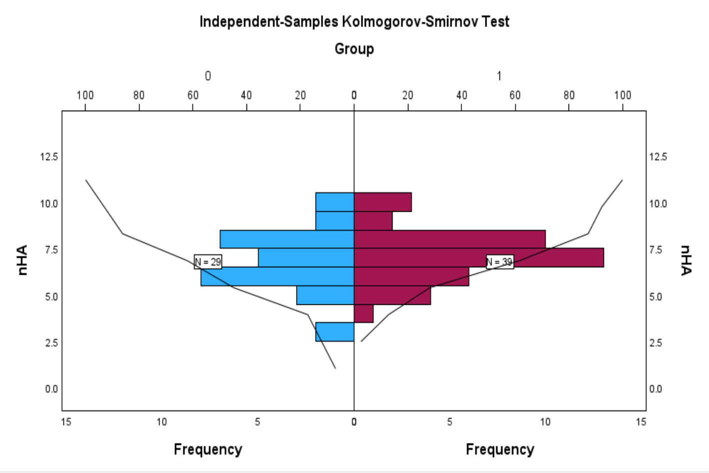
Independent-Samples Kolmogorov-Smirnov Test
Group
0
1
nHA
N = 29
N = 39
Frequency
Frequency

(f)

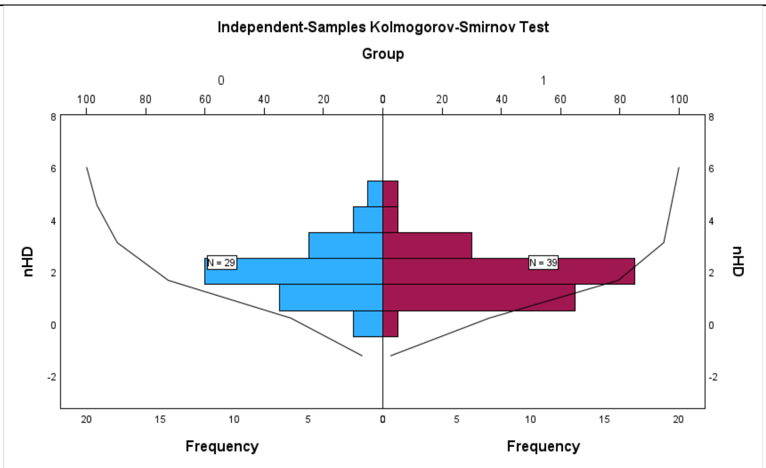

(g)

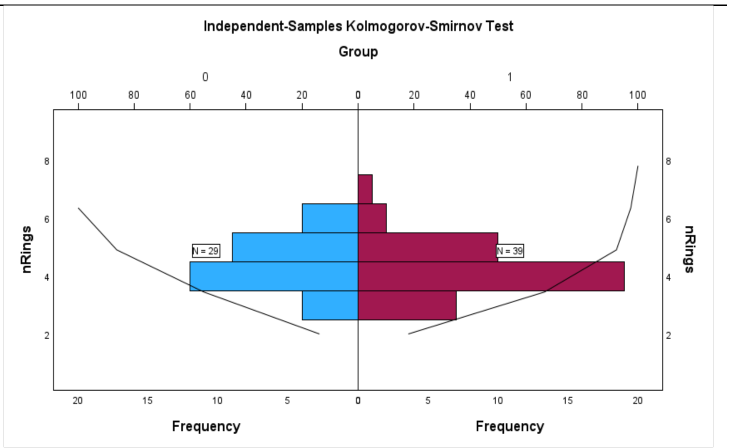

(h)

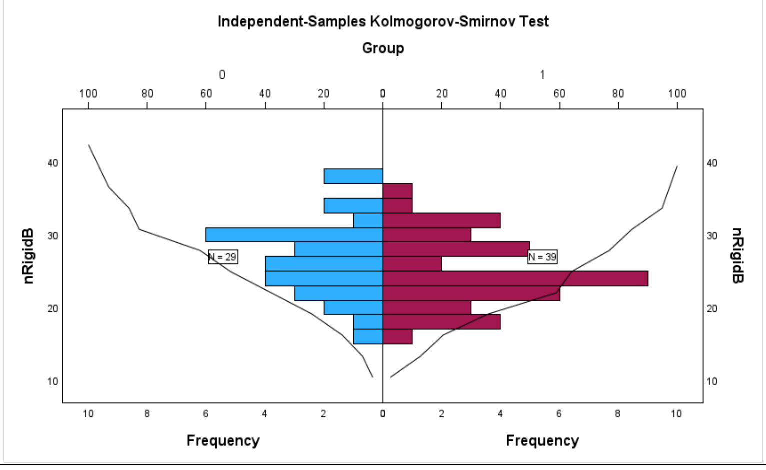

(i)

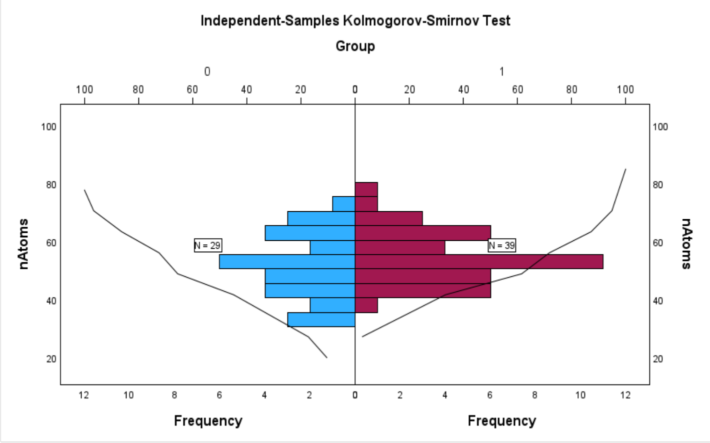

(j)

Figure S3. Bar graphical representations of Independent-Samples Kolmogorov-Smirnov Tests (**a-j**).

Table S6. The compounds in the top 10% ranked according to their CNN affinity.

	compound_id	true_label	affinity	pose_score	CNN_affinity
1	ZINC13495910	inactive	-8,67	0,9695	8,889
2	BDB27963	active	-10,51	0,9044	8,862
3	BDB27964	active	-9,66	0,8971	8,828
4	BDB27970	active	-8,64	0,9221	8,796
5	BDB5460	active	-10,47	0,9765	8,764
6	ZINC06443991	inactive	-10	0,943	8,735
7	BDB13921	active	-10,83	0,94	8,726
8	BDB5459	active	-10,03	0,9853	8,663
9	BDB27961	active	-11,93	0,9148	8,632
10	BDB13923	active	-10,88	0,9682	8,543
11	ZINC20911906	inactive	-7,35	0,7011	8,504
12	ZINC13845484	inactive	-7,16	0,8726	8,43
13	BDB5445	active	-11,24	0,9525	8,419
14	BDB5441	active	-10,7	0,9107	8,417
15	ZINC13494871	inactive	-8,31	0,9322	8,368
16	ZINC28132328	inactive	-10,15	0,9226	8,366
17	ZINC23740603	inactive	-9,49	0,9263	8,364
18	ZINC08758474	inactive	-9,37	0,9274	8,331
19	ZINC21852025	inactive	-6,2	0,7088	8,329
20	BDB5458	active	-9,7	0,9611	8,324
21	ZINC06073766	inactive	-5,17	0,8716	8,303
22	BDB13914	active	-11,49	0,9732	8,297
23	ZINC08826693	inactive	-10,31	0,9794	8,287
24	ZINC04739826	inactive	-9,27	0,8598	8,287
25	ZINC16749841	inactive	-7,59	0,8986	8,228
26	ZINC25801127	inactive	-7,12	0,7881	8,224
27	ZINC28848633	inactive	-9,38	0,9012	8,219
28	BDB13913	active	-11,29	0,9692	8,186
29	ZINC04685065	inactive	-1,39	0,6809	8,174
30	ZINC03340378	inactive	-7,58	0,9023	8,169
31	ZINC10176279	inactive	-8,22	0,7816	8,169
32	ZINC03376114	inactive	-8,91	0,921	8,161
33	ZINC26082335	inactive	-5,69	0,8682	8,161

Table S7. The compounds in the top 10% ranked according to their CNN pose score.

	compound_id	true_label	affinity	pose_score	CNN_affinity
1	BDB5459	active	-10,03	0,9853	8,663
2	BDB5447	active	-8,99	0,9816	8,01
3	ZINC08826693	inactive	-10,31	0,9794	8,287
4	BDB5460	active	-10,47	0,9765	8,764
5	ZINC28250348	inactive	-7,84	0,9761	8,144
6	BDB13914	active	-11,49	0,9732	8,297
7	ZINC09282440	inactive	-6,1	0,9724	8,066
8	BDB13902	active	-8,91	0,9713	7,869
9	ZINC19204006	inactive	-9,33	0,9696	8,071
10	ZINC13495910	inactive	-8,67	0,9695	8,889
11	BDB13913	active	-11,29	0,9692	8,186
12	ZINC08435259	inactive	-7,44	0,969	7,379
13	BDB5446	active	-7,84	0,9687	7,578
14	BDB13923	active	-10,88	0,9682	8,543
15	BDB13911	active	-11,88	0,9664	8,134
16	BDB13900	active	-8,18	0,9641	7,634
17	BDB13904	active	-11,23	0,9612	8,145
18	BDB5458	active	-9,7	0,9611	8,324
19	ZINC21327934	inactive	-9,49	0,9601	8,156
20	ZINC28951507	inactive	-9,84	0,9595	7,391
21	ZINC21326306	inactive	-11,59	0,959	7,281
22	ZINC04545643	inactive	2,47	0,9559	7,861
23	ZINC31916306	inactive	-9,64	0,9553	7,718
24	ZINC28895104	inactive	-7,42	0,9549	7,757
25	BDB5445	active	-11,24	0,9525	8,419
26	ZINC28253874	inactive	-7,14	0,952	7,957
27	BDB5456	active	-7,91	0,9472	8,059
28	BDB13903	active	-8,87	0,945	7,532
29	ZINC06443991	inactive	-10	0,943	8,735
30	ZINC09472352	inactive	-8,91	0,942	7,755
31	BDB13901	active	-8,06	0,9418	7,51
32	BDB5439	active	-9,11	0,9411	8,074
33	BDB5455	active	-9,92	0,9411	7,873

Table S8. Group Statistics of the Independent T-Tests.

Group Statistics											
**Group**		**N**	**Mean**	**Std. Deviation**	**Std. Error Mean**	**Group**		**N**	**Mean**	**Std. Deviation**	**Std. Error Mean**
**Molecular weight**	Studied compounds	29	438.92	99.11	18.40	**nHA**	Studied compounds	29	7	2	0
	FDA-approved drugs	39	439.14	68.90	11.03		FDA-approved drugs	39	7	1	0
**TPSA**	Studied compounds	29	94.96	24.77	4.60	**nHD**	Studied compounds	29	2	1	0
	FDA-approved drugs	39	89.65	19.05	3.05		FDA-approved drugs	39	2	1	0
**MR**	Studied compounds	29	123.94	26.77	4.97	**nRings**	Studied compounds	29	4	1	0
	FDA-approved drugs	39	121.90	20.84	3.34		FDA-approved drugs	39	4	1	0
**LogP**	Studied compounds	29	4.24	1.16	0.22	**nRigidB**	Studied compounds	29	26	6	1
	FDA-approved drugs	39	3.06	1.34	0.21		FDA-approved drugs	39	25	5	1
**nRB**	Studied compounds	29	6	3	1	**nAtoms**	Studied compounds	29	52	11	2
	FDA-approved drugs	39	6	2	0		FDA-approved drugs	39	55	9	1
